# Phenanthroline-Mediated Photoelectrical Enhancement in Calix[4]arene-Functionalized Titanium-Oxo Clusters

**DOI:** 10.3390/molecules29112566

**Published:** 2024-05-30

**Authors:** Jinle Hou, Chen Huang, Yuxin Liu, Pengfei Fei, Dongxu Zhang, Konggang Qu, Wenwen Zi, Xianqiang Huang

**Affiliations:** Shandong Provincial Key Laboratory of Chemical Energy Storage and Novel Cell Technology, School of Chemistry and Chemical Engineering, Liaocheng University, Liaocheng 252000, China; 19861527937@163.com (C.H.); 17861812770@163.com (Y.L.); 18863565739@163.com (P.F.); 15689054231@163.com (D.Z.); qukonggang@lcu.edu.cn (K.Q.); ziwenwen@lcu.edu.cn (W.Z.)

**Keywords:** titanium-oxo clusters, calix[n]arenes, crystal structure, photoelectric performance, DFT calculations

## Abstract

Incorporating two organic ligands with different functionalities into a titanium-oxo cluster entity simultaneously can endow the material with their respective properties and provide synergistic performance enhancement, which is of great significance for enriching the structure and properties of titanium-oxo clusters (TOCs). However, the synthesis of such TOCs is highly challenging. In this work, we successfully synthesized a TBC4A-functionalized TOC, [Ti_2_(TBC4A)_2_(MeO)_2_] (**Ti2**; MeOH = methanol, TBC4A = tert-butylcalix[4]arene). By adjusting the solvent system, we successfully introduced 1,10-phenanthroline (Phen) and prepared TBC4A and Phen co-protected [Ti_2_(TBC4A)_2_(Phen)_2_] (**Ti2-Phen**). Moreover, when Phen was replaced with bulky 4,7-diphenyl-1,10-phenanthroline (Bphen), [Ti_2_(TBC4A)_2_(Bphen)_2_] (**Ti2-Bphen**), which is isostructural with **Ti2-Phen**, was obtained, demonstrating the generality of the synthetic method. Remarkably, **Ti2-Phen** demonstrates good stability and stronger light absorption, as well as superior photoelectric performance compared to **Ti2**. Density functional theory (DFT) calculations reveal that there exists ligand-to-core charge transfer (LCCT) in **Ti2**, while an unusual ligand-to-ligand charge transfer (LLCT) is present in **Ti2-Phen**, accompanied by partial LCCT. Therefore, the superior light absorption and photoelectric properties of **Ti2-Phen** are attributed to the existence of the unusual LLCT phenomenon. This study not only deeply explores the influence of Phen on the performance of the material but also provides a reference for the preparation of materials with excellent photoelectric performance.

## 1. Introduction

Titanium-oxo clusters (TOCs) have gained significant attention in recent years due to their fascinating structures and potential applications in photocatalytic water splitting [1,2,3], solar cells [4,5,6], photocatalytic degradation of pollutants [7,8,9], photocatalytic organic synthesis [10,11,12,13], CO_2_ reduction [14,15], and other areas [16,17]. The driving force behind TOC research lies in their ability to serve as structural and reactivity models for TiO_2_ materials. TOCs with atomically precise structural information are crucial for establishing structure–property relationships at the molecular level [18,19,20]. Furthermore, as metal aggregates, TOCs can generate abundant metal active sites by precisely controlling the coordination environment of metal ions, enabling their application as crystalline catalysts in a wide range of photocatalytic reactions. Over the past decade, the development of numerous synthetic strategies has led to the synthesis of a diverse array of TOCs with varying structures and properties, propelling the advancement of the TOCs [21,22,23,24,25]. Despite these advancements, most TOCs suffer from poor stability due to the presence of alkoxy groups (-OR) from the solvent or Ti(OR)_4_ on their periphery. These alkoxy groups typically exhibit monodentate or bridging coordination modes, resulting in insufficient protection of the titanium-oxo core. Another limitation of TOCs, besides poor stability, is that their light absorption is primarily confined to the ultraviolet region, hindering the effective utilization of sunlight. Ligand modification strategies have emerged as a promising approach to enhance the light absorption capability and improve the stability of TOCs [26,27,28,29]. Therefore, the rational selection of functional ligands is paramount for the synthesis of novel TOCs with narrow bandgaps and outstanding stability.

Ligands used to assemble TOCs are mainly concentrated in carboxylates, phosphonates, catechols, quinolines, oximes, and their combinations [30,31,32]. Recently, our group successfully synthesized a black TOC fully protected by catechol ligands, which greatly expands its absorption range and improves its stability [33]. Beyond these conventional ligands, calix[n]arenes, which consist of multiple phenol units linked by methylene bridges at the ortho-position, exhibit a cavity structure and are generally considered to be the third generation of supramolecules [34,35]. Due to the unique properties of calix[n]arenes, including multiple coordination sites, variable spatial configurations, and diverse coordination modes, they have become an ideal choice for constructing novel metal nanoclusters. The Liao group and Sun group have made outstanding achievements in the field of calix[n]arene-metal clusters, including Co_32_, Ni_40_, Cu_12_, and Ag_155_ [36,37,38,39]. However, there are few reports on atomically precise calix[n]arene-protected TOCs [40,41,42,43,44]. Recently, Lan’s group successfully synthesized calix[4]arene-functionalized TOCs, providing an important reference for the application of calix[n]arenes in the assembly of TOCs [45]. Therefore, the synthesis of TOCs protected by macrocyclic ligands such as calix[n]arene is urgently needed, as it will help to reveal key chemical fundamentals, such as the stacking mode of metal atoms and the metal–ligand interface pattern.

In addition, N-doping has been shown to have a significant impact on the properties of TiO_2_ [46,47], but TOCs functionalized with N-containing ligands, such as analogs, are relatively rare [48,49]. Due to the relatively weak affinity between N atoms and Ti, in previous reports, bifunctional ligands containing both N and O atoms were typically introduced into the synthetic system of TOCs [50,51]. Recently, 1,10-phenanthroline (Phen), as a typical bidentate nitrogen donor ligand and π-conjugated chromophore, has been employed to construct functional TOCs [52,53,54,55,56]. For instance, the Xuan group successfully synthesized phen-functionalized cocrystal TOCs and achieved the separation of pure-phase crystals by adjusting the synthesis conditions, which were then successfully applied to the photocatalytic sulfoxidation reaction [57]. Nevertheless, the structural library of phen-functionalized TOCs remains limited.

Based on the above considerations, the simultaneous incorporation of calix[n]arene and phen ligands into a TOC entity would endow the material with both their individual properties and synergistically enhanced performance, thereby enriching the structural diversity and performance of the TOCs. However, the synthesis of such TOCs is challenging due to the large steric hindrance of these ligands. Herein, we successfully synthesized a TBC4A-functionalized TOC, [Ti_2_(TBC4A)_2_(MeO)_2_] (**Ti2**; MeOH = Methanol, TBC4A = tert-butylcalix[4]arene). By tuning the solvent system, we successfully introduced 1,10-phenanthroline (Phen), leading to the formation of TBC4A and Phen co-protected [Ti_2_(TBC4A)_2_(Phen)_2_] (**Ti2-Phen**). In addition, in the synthetic system of **Ti2-Phen**, when Phen is replaced with the bulky 4,7-diphenyl-1,10-phenanthroline (Bphen), a **Ti2-Phen** analog, [Ti_2_(TBC4A)_2_(Bphen)_2_] (**Ti2-Bphen**), is obtained, confirming the general applicability of this synthetic method. Density functional theory (DFT) calculations revealed that an unusual ligand-to-ligand charge transfer (LLCT) phenomenon occurred in **Ti2-Phen**, from the TBC4A ligand to the Phen ligand. This LLCT phenomenon leads to a broader light absorption range for **Ti2-Phen** than **Ti2**, thus endowing **Ti2-Phen** with superior photoelectric properties compared to **Ti2**.

## 2. Results and Discussion

### 2.1. Syntheses and Characterization of Ti2, Ti2-Phen, and Ti2-Bphen

**Scheme 1:**
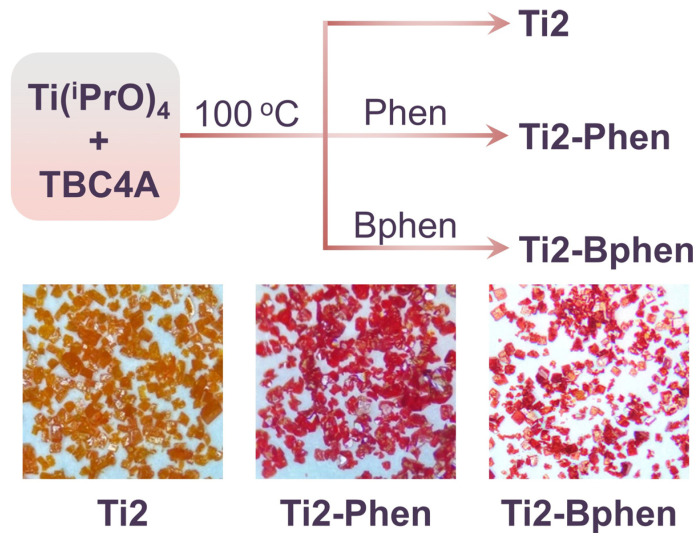
Schematic diagram of the synthesis for **Ti2**, **Ti2-Phen**, and **Ti2-Bphen**. (TBC4A = tert-butylcalix[4]arene; ^i^PrOH = isopropanol; Phen = 1,10-phenanthroline; Bphen = 4,7-diphenyl-1,10-phenanthroline).

The crystals of **Ti2**, **Ti2-Phen,** and **Ti2-Bphen** were all synthesized using a one-pot method with moderate yield (Scheme 1). Briefly, Ti(^i^PrO)_4_ and H_4_TBC4A were dissolved in a binary solvent of DMF and methanol (*v*:*v* = 2:3) at room temperature. The resulting mixture was then reacted at 100 °C for 5 days and subsequently cooled to room temperature, yielding yellow crystals of **Ti2**. Considering the significant influence of N-containing ligands on the structure and properties of TOCs [47], we aimed to introduce such ligands into TBC4A-functionalized TOCs to improve their structure and performance. Due to the highly conjugated π-electron system and strong coordination ability to titanium atoms, Phen was chosen as an ideal ligand. Consequently, we directly added Phen to the synthesis system of **Ti2**. Unfortunately, instead of crystals, we obtained precipitates. Given the importance of solvents in the crystallization of metal clusters [58], we replaced the binary solvent in the synthesis system of **Ti2** with isopropanol and introduced Phen. Excitingly, we successfully synthesized red crystals of **Ti2-Phen**. We attempted to lower the temperature to 80 °C and change the type of solvent. The results indicate that temperature and solvent are crucial for the synthesis of **Ti2-Phen** (Table S1). Furthermore, under the conditions of synthesizing **Ti2-Phen**, we further replaced Phen with Bphen with larger steric hindrance, keeping other synthesis conditions unchanged, and successfully obtained red crystals of **Ti2-Bphen**, demonstrating the universality of the synthesis scheme. With consistent experimental parameters for **Ti2**, **Ti2-Phen**, and **Ti2-Bphen**, their reproducibility is quite good. The synthesis details and X-ray diffraction data for **Ti2**, **Ti2-Phen**, and **Ti2-Bphen** are presented in the Materials and Methods section, along with Table S2.

The powder X-ray diffraction (PXRD) patterns of **Ti2**, **Ti2-Phen**, and **Ti2-Bphen** exhibit high consistency with simulation results derived from crystallographic data (Figures S1–S3). This strongly suggests their high crystallinity and phase purity. The infrared (IR) spectra of **Ti2**, **Ti2-Phen**, and **Ti2-Bphen** are shown in Figure S4. The presence of Phen or Bphen ligands was confirmed by the vibrational peaks in the 3000–3100 cm^−1^ range. In addition, the tert-butyl groups of the calixarenes were characterized by C-H vibrational peaks at 2960 cm^−1^. The vibrational bands around 1000 cm^−1^ were assigned to Ti-O-C vibrations. The elemental mapping diagrams for **Ti2**, **Ti2-Phen**, and **Ti2-Bphen** are presented in Figures S5–S7, demonstrating the presence of Ti, C, O, and N elements. This further corroborates the purity of the synthesized samples. To ascertain the chemical composition and elemental states of **Ti2**, **Ti2-Phen**, and **Ti2-Bphen**, X-ray photoelectron spectroscopy (XPS) was conducted. As depicted in Figures S8–S10, the Ti 2p spectra distinctly exhibit two peaks around 458 eV and 464 eV, corresponding to the states of Ti 2p_3/2_ and Ti 2p_1/2_, respectively. This indicates the presence of only Ti^4+^ in the clusters **Ti2**, **Ti2-Phen**, and **Ti2-Bphen**.

### 2.2. Structure Analyses of Ti2, Ti2-Phen, and Ti2-Bphen

**Figure 1:**
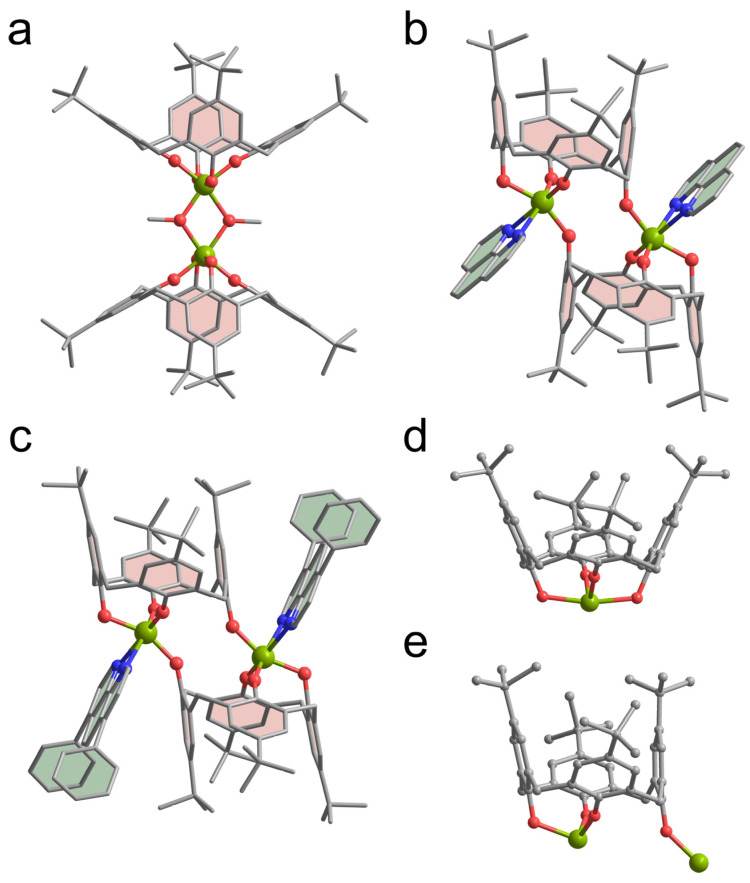
(**a**) Crystal structure of **Ti2**. (**b**) Crystal structure of **Ti2-Phen**. (**c**) Crystal structure of **Ti2-Bphen**. (**d**) The coordination mode of TBC4A in **Ti2**. (**e**) The coordination mode of TBC4A in **Ti2-Phen**. Hydrogen atoms have been omitted for clarity. Color code: green for Ti, red for O, gray for C, and blue for N.

Single crystal X-ray diffraction (SCXRD) analysis reveals that **Ti2** crystallizes in the triclinic space group *P*-1, with its asymmetric unit containing half of a cluster. **Ti2** consists of two titanium atoms, two TBC4A ligands, and two methanol molecules (Figure 1a). All titanium atoms in **Ti2** adopt a distorted octahedral coordination geometry. Specifically, each Ti atom coordinates with 4 O atoms from TBC4A ligands and 2 O atoms from two methanol molecules. The outer space of **Ti2** is surrounded by one TBC4A ligand above and one TBC4A ligand below, along with two methanol molecules exhibiting a bridging coordination mode. Consequently, **Ti2** can be viewed as a {Ti_2_} core sandwiched between two TBC4A ligands, forming a sandwich-shaped structure. Additionally, both TBC4A ligands exhibit the same coordination mode of μ_1_-η^1^:η^1^:η^1^:η^1^ (Ti-O = 1.79–2.03 Å) (Figure 1d).

It is worth noting that the **Ti2** and **Ti2-MeOH** reported by Liu [59] possess the same skeletal structure. However, they crystallize in different crystal systems with different space groups (**Ti2-MeOH** in monoclinic space group *P*2_1_/n and **Ti2** in triclinic space group *P*-1) (Table S3). In addition, during the synthesis process, we utilized different reactant ratios, solvents, reaction times, and temperatures compared to **Ti2-MeOH** (Table S4). Consequently, **Ti2** differs from **Ti2-MeOH**, and they can be regarded as polymorphs. Polymorphs often display distinct properties due to different crystalline forms, which are crucial for the study of metal clusters [60]. A further detailed comparison of the physical properties of **Ti2** and **Ti2-MeOH** reveals that despite their different crystal appearances resulting from different crystallization forms, they exhibit similar TGA results.

SCXRD reveals that **Ti2-Phen** crystallizes in a monoclinic system with the space group *C*2/c, and its asymmetric unit contains two independent half-clusters. In contrast, **Ti2-Bphen** crystallizes in a monoclinic system with the space group *C*2/m, and its asymmetric unit contains two independent quarter-clusters. Given the isostructural nature of **Ti2-Phen** and **Ti2-Bphen** (Figure 1b,c), we will focus our analysis on the structure of **Ti2-Phen**. **Ti2-Phen** consists of two titanium atoms, two TBC4A ligands, and two Phen ligands. All titanium atoms in **Ti2-Phen** adopt a distorted octahedral coordination configuration. Specifically, each Ti atom coordinates with three O atoms from one TBC4A ligand, one O atom from another TBC4A ligand, and two N atoms from one Phen ligand (Figure 1b). From a structural perspective, the two Ti atoms are directly connected by two TBC4A ligands to form a {TBC4A-Ti_2_} unit, presenting a chair-like configuration. Additionally, the periphery of the {TBC4A-Ti_2_} unit is chelated by two Phen ligands (Ti-N = 2.272–2.278 Å), further stabilizing the overall cluster structure. Interestingly, both TBC4A ligands adopt a μ_2_-η^1^:η^1^:η^1^:η^1^ coordination mode (Ti-O = 1.83–1.96 Å) (Figure 1e), unlike the TBC4A ligands in **Ti2**. It is worth noting that the two Ti atoms within **Ti2-Phen** are spatially isolated, with no O atoms bridging between them. Consequently, the **Ti2-Phen** features small voids.

By comprehensively analyzing the crystal structures of **Ti2** and **Ti2-Phen**, we found that they share the same number of TBC4A ligands and Ti atoms, despite having different structural configurations and distinct coordination modes of TBC4A. Compared to **Ti2**, **Ti2-Phen** introduces an additional Phen functional ligand. Therefore, **Ti2** and **Ti2-Phen** can serve as model compounds for studying the influence of Phen ligands on the properties of the material.

### 2.3. Stability of Ti2, Ti2-Phen, and Ti2-Bphen

**Figure 2:**
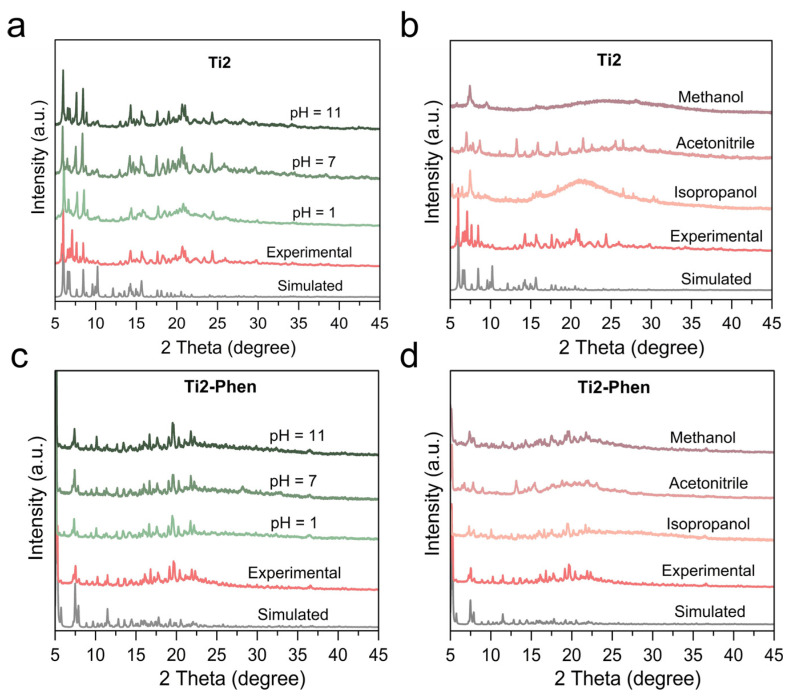
(**a**) PXRD patterns of **Ti2** immersed in water solutions with pH values of 1, 7, and 11 for 24 h. (**b**) PXRD patterns of **Ti2** immersed in common organic solvents for 24 h. (**c**) PXRD patterns of **Ti2-Phen** immersed in water solutions with pH values of 1, 7, and 11 for 24 h. (**d**) PXRD patterns of **Ti2-Phen** immersed in common organic solvents for 24 h.

The stability of metal nanoclusters is crucial for accurately assessing their catalytic performance and exploring their potential applications in industry [61,62]. Generally, partial regions on the surface of many TOCs are encapsulated by alkoxide ligands, resulting in poor stability in air or aqueous conditions [51]. Subsequently, to evaluate the chemical stability of **Ti2**, **Ti2-Phen**, and **Ti2-Bphen**, these clusters were immersed in aqueous solutions with pH values of 1, 7, and 11 for 24 h. The results show that the PXRD patterns before and after immersion are roughly consistent, indicating that these three TOCs can maintain structural integrity across a wide pH range (Figure 2a,c and Figure S11). Despite exhibiting good pH stability, **Ti2** shows instability when immersed in different solvents such as isopropanol, acetonitrile, and methanol, as evidenced by changes in its PXRD patterns (Figure 2b). This is because the surface of **Ti2** has two weakly coordinated methanol ligands that are prone to detachment, exposing Ti sites and rendering them unstable. Interestingly, **Ti2-Phen** exhibits excellent solvent stability as evidenced by the nearly identical PXRD patterns before and after immersion in common solvents such as isopropanol, acetonitrile, and methanol (Figure 2d).

Additionally, the thermal stability of **Ti2**, **Ti2-Phen**, and **Ti2-Bphen** was evaluated through thermogravimetric analysis (TGA) (Figure S12). The results indicate that they can maintain structural integrity at approximately 140 °C, 412 °C, and 360 °C, respectively. Specifically, the mass loss of **Ti2** below 160 °C corresponds to the loss of two methanol molecules and one DMF molecule. Following this, the TBC4A ligands in **Ti2** begin to lose around 500 °C and are subsequently completely converted to metal oxides. **Ti2-Phen** and **Ti2-Bphen** begin to lose Phen or Bphen ligands around 410 °C and 360 °C, respectively, and then lose TBC4A ligands around 490 °C, eventually converting to metal oxides.

Compared to **Ti2**, **Ti2-Phen** demonstrated notable acid–base stability, organic solvent stability, and thermal stability, which can be attributed to the introduction of the Phen ligand. This exceptional stability makes **Ti2-Phen** a promising candidate for heterogeneous catalysis in water/organic solvent media as well as under high-temperature conditions.

### 2.4. Photoelectric Properties of Ti2, Ti2-Phen, and Ti2-Bphen

**Figure 3:**
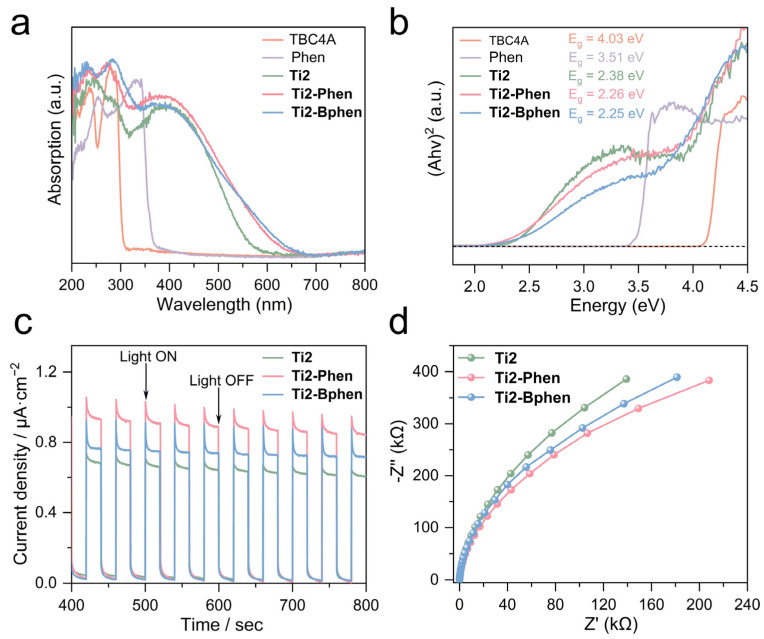
(**a**) Solid-state UV-visible absorption spectra of TBC4A, Phen, **Ti2**, **Ti2-Phen**, and **Ti2-Bphen**. (**b**) Tauc plots of TBC4A, Phen, **Ti2**, **Ti2-Phen**, and **Ti2-Bphen**. (**c**) Transient photocurrent responses of **Ti2**, **Ti2-Phen**, and **Ti2-Bphen** under Xe lamp irradiation. (**d**) Electrochemical impedance spectroscopy (EIS) Nyquist plots of **Ti2**, **Ti2-Phen**, and **Ti2-Bphen**.

To assess the light absorption capabilities of **Ti2**, **Ti2-Phen**, and **Ti2-Bphen**, solid-state UV-visible absorption spectra were tested. Additionally, for comparison, we also tested the light absorption ranges of TBC4A and Phen ligands (Figure 3a). The results clearly showed that both TBC4A and Phen ligands exhibited narrow light absorption ranges with absorption edges of 300 nm and 370 nm, respectively. As expected, TBC4A-functionalized **Ti2** exhibited a wide light absorption range with an absorption edge of 570 nm. Excitingly, **Ti2-Phen,** co-protected by TBC4A and Phen, exhibited an even wider light absorption range with an absorption edge extended to 660 nm. In addition, **Ti2-Bphen** exhibited a light absorption range similar to that of **Ti2-Phen**. It is generally believed that the UV light absorption band of traditional TOCs is mainly caused by the O→Ti charge transfer in the titanium-oxo core [31]. A comprehensive analysis of the experimental results leads to the following conclusions: (1) The introduction of TBC4A ligands can significantly change the light absorption properties of TOCs. (2) In the presence of TBC4A ligands, the introduction of Phen can further broaden the light absorption range of TOCs. In addition, previously reported Phen-functionalized TOCs are colorless and have light absorption limited to the ultraviolet region [56,57]. Therefore, the wider light absorption range of **Ti2-Phen** is attributed to the synergistic effect of TBC4A and Phen. Based on the Kubelka–Munk function (αh*υ*)^2^ = *κ*(h*υ*−E_g_) (where E_g_ is the bandgap (eV), h is Planck’s constant (J·s), *υ* is the light frequency (s^−1^), *κ* is the absorption coefficient, and α is the absorption coefficient), the optical bandgaps of **Ti2**, **Ti2-Phen**, and **Ti2-Bphen** are estimated to be 2.38, 2.26, and 2.25 eV, respectively (Figure 3b). These results indicate that these materials exhibit semiconductor-like properties.

In order to evaluate the efficiency of **Ti2**, **Ti2-Phen**, and **Ti2-Bphen** in separating photogenerated electron–hole pairs, we conducted transient short-circuit photocurrent measurements. The test system was composed of a TOC-modified indium tin oxide (ITO) as the working electrode, Ag/AgCl as the reference electrode, and a platinum wire as the counter electrode. These tests were carried out in a 0.20 mol·L^−1^ Na_2_SO_4_ electrolyte solution under irradiation of a 150 W high-pressure xenon lamp, and the applied bias potential was maintained at 0.6 V. Under xenon lamp irradiation, the photocurrent density of all samples significantly increased, and upon cessation of lamp irradiation, their photocurrent density rapidly decreased (Figure 3c). This finding indicates that **Ti2**, **Ti2-Phen**, and **Ti2-Bphen** all exhibit significant charge separation efficiency under illumination conditions. Specifically, the order of photocurrent densities is **Ti2-Phen** (0.92 μA·cm^−2^) > **Ti2-Bphen** (0.76 μA·cm^−2^) > **Ti2** (0.66 μA·cm^−2^). The photocurrent densities of **Ti2-Phen** and **Ti2-Bphen**, both protected by dual ligands, are higher than that of **Ti2** protected by TBC4A, indicating their capability to capture more photogenerated electrons and more effectively suppress the recombination of electrons and holes. Furthermore, the stability of **Ti2-Phen** after photocurrent testing was investigated. As shown in Figure S13, the PXRD pattern of **Ti2-Phen** after photocurrent testing is essentially the same as before the reaction, indicating that **Ti2-Phen** did not decompose during the photocurrent measurement. To assess the long-term stability of the **Ti2-Phen** sample under continuous light exposure, we conducted six cycles of photocurrent testing, with each cycle lasting 10 min (Figure S14). Excitingly, after the six cycles of experimentation, the photocurrent density of the sample remained largely unchanged, demonstrating the photostability of **Ti2-Phen**. To further understand the charge transfer processes of **Ti2**, **Ti2-Phen**, and **Ti2-Bphen**, electrochemical impedance spectroscopy (EIS) tests were conducted. As shown in Figure 3d, the Nyquist plot of **Ti2-Phen** is smaller than that of **Ti2** and **Ti2-Bphen**, indicating that the interfacial charge transfer process of **Ti2-Phen** is faster [63], consistent with the photocurrent measurement results.

### 2.5. Theoretical Calculation of Ti2 and Ti2-Phen

**Figure 4:**
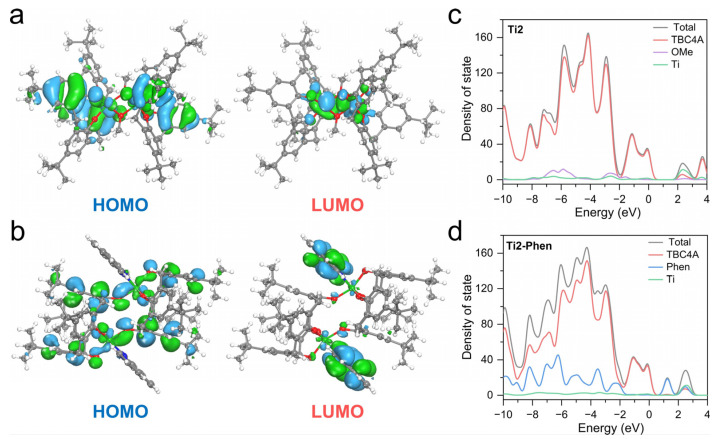
(**a**) Molecular orbital distribution of HOMO and LUMO for **Ti2** based on DFT calculations. (**b**) Molecular orbital distribution of HOMO and LUMO for **Ti2-Phen** based on DFT calculations. (**c**) DOS plot for **Ti2** based on DFT calculations. (**d**) DOS plot for **Ti2-Phen** based on DFT calculations.

As reported previously, introducing functional ligands into TOCs can broaden the range of visible light absorption due to the generation of ligand-to-metal charge transfer or metal-to-ligand charge transfer [29,33,44]. To investigate the charge transfer properties of **Ti2** and **Ti2-Phen** in an attempt to explain their differences in optical properties, we performed theoretical density functional theory (DFT) calculations of **Ti2** and **Ti2-Phen**. As shown in Figure 4a,b, atomic orbital (AO) characteristic analysis of the frontier orbital compositions of **Ti2** and **Ti2-Phen** is provided. Theoretical analysis results show that the electron density of the highest occupied molecular orbitals (HOMOs) of **Ti2** and **Ti2-Phen** is mainly located on the TBC4A ligand, but the electron density distribution of their lowest unoccupied molecular orbitals (LUMOs) is different. For **Ti2**, LUMOs are mainly dominated by the Ti 3d orbitals of the titanium-oxo core, while for **Ti2-Phen**, LUMOs are mainly distributed on the Phen ligand and partially distributed on the titanium-oxo core. In addition, the density of states (DOS) plot of **Ti2** clearly shows that its valence band is mainly dominated by the TBC4A ligand, and the conduction band is located on the titanium-oxo core (Figure 4c). However, the DOS plot of **Ti2-Phen** shows that its valence band is located on the TBC4A ligand, and the conduction band is jointly controlled by Phen and the titanium-oxo core (Figure 4d). Based on the above results, it can be concluded that there is a ligand-to-core charge transfer (LCCT) in **Ti2**, while there is a ligand-to-ligand charge transfer (LLCT) in **Ti2-Phen**, accompanied by partial LCCT. It is worth noting that the reports of LLCT in TOCs are very rare [64]. Therefore, the excellent light absorption and photoelectric properties of **Ti2-Phen** are attributed to the existence of an unusual LLCT phenomenon.

## 3. Materials and Methods

### 3.1. Materials

Titanium (IV) tetraisopropanolate (Ti(^i^PrO)_4_, 98%, Adamas-beta^®^, Shanghai, China), tert-butylcalix[4]arene (TBC4A), 1,10-phenanthroline (Phen), and 4,7-diphenyl-1,10-phenanthroline (Bphen) were purchased from Shanghai Titan Scientific Co., Ltd. (Shanghai, China) Methanol (MeOH, 99.7%), DMF (99.7%), ethanol (EtOH, 99.7%), and isopropanol (^i^PrOH, 99.7%) were purchased from Sinopharm Chemical Reagent Co., Ltd., Shanghai, China. All chemicals and solvents were of analytical grade and used without further purification.

### 3.2. Preparation of Ti2, Ti2-Phen, and Ti2-Bphen

Synthesis of [Ti_2_(TBC4A)_2_(OMe)_2_] (**Ti2**): 0.10 mmol (64.9 mg) of tert-butylcalix[4]arene was ultrasonically dissolved in a 5 mL binary solvent of DMF and methanol (*v*:*v* = 2:3). Subsequently, 0.66 mmol (0.20 mL) of Ti(^i^PrO)_4_ was added, and the mixture was sonicated for an additional 20 min. Finally, the mixture was sealed in a 10 mL glass vial and heated at 100 °C for 5 days. Upon cooling to room temperature, yellow crystals were obtained by filtration and washed several times with ethanol. Yield: 20 mg.

Synthesis of [Ti_2_(TBC4A)_2_(Phen)_2_] (**Ti2-Phen**): The mixture of 0.10 mmol (64.9 mg) of tert-butylcalix[4]arene and 0.30 mmol (54 mg) of 1,10-phenanthroline was ultrasonically dissolved in 5 mL of isopropanol. Subsequently, 0.66 mmol (0.20 mL) of Ti(^i^PrO)_4_ was added, and the mixture was sonicated for an additional 20 min. Finally, the mixture was sealed in a 10 mL glass vial and heated at 100 °C for 3 days. Upon cooling to room temperature, red crystals were obtained by filtration and washed several times with isopropanol. Yield: 15 mg.

Synthesis of [Ti_2_(TBC4A)_2_(Bphen)_2_] (**Ti2-Bphen**): Similar to **Ti2-Phen**, except that 1,10-phenanthroline (0.30 mmol, 54 mg) was replaced with 4,7-diphenyl-1,10-phenanthroline (0.30 mmol, 100 mg). The other conditions were kept the same. After cooling to room temperature, red crystals were obtained by filtration and washed several times with isopropanol. Yield: 18 mg.

### 3.3. Characterization of Materials

Powder X-ray diffraction (PXRD) data were carried out on a microcrystalline powdered sample using a Rigaku SmartLab-9Kw (Rigaku Corporation, Tokyo, Japan) diffractometer using Cu radiation (λ = 1.54184 Å). Thermogravimetry (TG) analysis was performed on a STA449F5/QMS403D instrument (Mettler-Toledo, Schwerzenbach, Switzerland) with a heating rate of 10 °C min^−1^ from 20 to 800 °C in N_2_ flow. The solid-state UV/Vis spectra data of the cluster samples were obtained using a Carry 500 UV-VIS spectrophotometer (Agilent Technologies, Santa Clara, CA, USA) with a scanning wavelength range from 200 nm to 800 nm. Fourier transform infrared spectroscopy (FTIR) measurements were obtained using a Nicolet iS50 spectrophotometer (Thermo Fisher Scientific, Waltham, MA, USA). The X-ray photoelectron spectroscopy (XPS) spectra were collected on a ESCALAB Xi^+^ instrument (Thermo Fisher Scientific, USA). The element mapping of samples was acquired on a FIB-SEM-GX4 scanning electron microscope (Thermo Fisher Scientific, USA). Photoelectrochemical measurements were carried out on CHI 660E electrochemical workstation (Chenhua, Shanghai, China) in a standard three-electrode electrochemical cell with ITO coated with the crystals as the working electrode, a platinum plate as counter electrode, and a saturated Ag/AgCl electrode as reference electrode. A sodium sulfate solution (0.2 M) was used as the electrolyte, and a Xe lamp (150 W) was used as the light source. A bias potential of 0.6 V was maintained. Preparation of the working electrode: 2 mg crystals powder was mixed with 0.99 mL ethanol and 10 μL Nafion D-520 dispersion solutions and sonicated for 30 min. Subsequently, 200 μL of slurry was transferred, coated on ITO glass plates (1 cm × 2 cm), and then dried at room temperature. Electrochemical impedance spectra (EIS) measurements were also carried out on CHI 660E electrochemical workstation via a conventional three-electrode system with a working electrode, a platinum plate as counter electrode, and a saturated Ag/AgCl electrode as reference electrode in a 0.2 M Na_2_SO_4_ aqueous solution over a frequency range of 100 kHz–0.01 Hz.

### 3.4. Single-Crystal X-ray Diffraction

Single crystals of **Ti2**, **Ti2-Phen**, and **Ti2-Bphen** were selected under an optical microscope and rapidly coated with high vacuum grease (Dow Corning Corporation) to prevent decomposition. The single-crystal diffraction analysis of **Ti2** was conducted using a Bruker SMART CCD diffractometer with Mo-Kα radiation (λ = 0.71073 Å) at 293 K. However, the single-crystal diffraction analysis of **Ti2-Phen** and **Ti2-Bphen** were recorded on Bruker D8 VENTURE diffractometer with an Incoatec I*μ*S 3.0 Cu EF (Incoatec IμS diamond Mo) microfocus source (55 W, Cu Kα, λ = 1.54184 Å) at 173 K equipped with a PHOTON III C28 detector and an Oxford Cryosystems CryostreamPlus 800 open-flow N_2_ cooling device. These structures were solved by the inherent phase method in the SHELXT program and refined by full-matrix least-squares techniques against *F*^2^ using the SHELXL program through the OLEX2 interface. Hydrogen atoms in carbon were placed in calculated positions and refined isotropically by using a riding model. Appropriate restraints or constraints were applied to the geometry and the atomic displacement parameters of the atoms in the cluster. All structures were examined using the Addsym subroutine of PLATON to ensure that no additional symmetry could be applied to the models. Pertinent crystallographic data collection and refinement parameters are collated in Table S1. The crystallographic data for **Ti2**, **Ti2-Phen**, and **Ti2-Bphen** were delivered to the Cambridge Crystallographic Data Centre (CCDC) with No. 2,349,973 for **Ti2**, No. 2,349,974 for **Ti2-Phen** and 2,349,975 for **Ti2-Bphen**. These data can be obtained from the CCDC via www.ccdc.cam.ac.uk/data_request/cif (accessed on 22 April 2024).

### 3.5. Computational Studies

The geometric structures of **Ti2** and **Ti2-Phen** were optimized by using Perdew–Burke–Ernzerhof (PBE) exchange–correlation functional in DMol^3^. The DFT-based relativistic semi-core pseudopotential (DSPP) and double numerical plus *d*-functions (DND) basis sets were used to calculate the energy of structural. The convergence criterion of the geometric optimization and electronic structure calculation was set to be 1.0 × 10^−5^ Hartree for energy change, 2.0 × 10^−3^ Hartree/Å for the gradient, and 3.0 × 10^−3^ Å for the displacement, respectively. Dispersion corrections in Grimme’s scheme (DFT-D3) were applied to treat the long-range van der Waals interactions.

## 4. Conclusions

In summary, by adjusting the solvent system, we successfully synthesized three atomically precise TOCs, **Ti2**, **Ti2-Phen**, and **Ti2-Bphen**. It is worth noting that **Ti2** and **Ti2-Phen** share the same number of TBC4A ligands and Ti atoms, with **Ti2-Phen** additionally incorporating Phen functional ligands. Consequently, **Ti2** and **Ti2-Phen** serve as model compounds for investigating the influence of Phen ligands on material properties. Notably, **Ti2-Phen** demonstrates good stability and a broader light absorption spectrum, as well as superior photoelectric performance compared to **Ti2**. Density functional theory (DFT) calculations revealed the presence of an unusual ligand-to-ligand charge transfer (LLCT) phenomenon in **Ti2-Phen**, which is absent in **Ti2**. Hence, the enhanced light absorption and photoelectric performance of **Ti2-Phen** stem from this unique LLCT phenomenon. This research not only serves as a valuable reference for the development of new materials boasting outstanding photoelectric properties but also paves the way for the application of these TOCs in practical devices or systems. For instance, the strong light absorption and efficient charge transfer properties of **Ti2-Phen** suggest its potential use in solar cells or photodetectors. Further research efforts could explore strategies to integrate these TOCs into functional devices and optimize their performance for real-world applications.

## Data Availability

Data are contained within the article and Supplementary Materials.

## Supplementary Materials

The following supporting information can be downloaded at: https://www.mdpi.com/article/10.3390/molecules29112566/s1, Figure S1. The compared PXRD patterns of **Ti2**. Figure S2. The compared PXRD patterns of **Ti2-Phen**. Figure S3. The compared PXRD patterns of **Ti2-BPhen**. Figure S4. The Fourier transform infrared spectroscopy (FT-IR) of **Ti2**, **Ti2-Phen**, and **Ti2-Bphen**. Figure S5. Elemental mapping images of Ti, O, and C of selected area for **Ti2**. Figure S6. Elemental mapping images of Ti, O, and N of selected area for **Ti2-Phen**. Figure S7. Elemental mapping images of Ti, O, and N of selected area for **Ti2-Bphen**. Figure S8. X-ray photoelectron spectroscopy (XPS) spectra of **Ti2**. Figure S9. X-ray photoelectron spectroscopy (XPS) spectra of **Ti2-Phen**. Figure S10. X-ray photoelectron spectroscopy (XPS) spectra of **Ti2-Bphen**. Figure S11. PXRD patterns of **Ti2-Bphen** immersed in water solutions with pH values of 1, 7, and 11 for 24 h. Figure S12. The thermogravimetric analysis (TGA) curves of **Ti2**, **Ti2-Phen**, and **Ti2-Bphen**. Figure S13. The PXRD patterns of **Ti2-Phen** before and after photocurrent testing. Figure S14. The six cycles of photocurrent testing of **Ti2-Phen**. Table S1. Exploration of the synthesis of **Ti2-Phen**. Table S2. Crystal data collection and structure refinement for **Ti2**, **Ti2-Phen,** and **Ti2-Bphen**. Table S3. Comparison of crystallographic parameters for **Ti2** and **Ti2-MeOH.** Table S4. Comparison of synthesis details for **Ti2** and **Ti2-MeOH**.
