# Supplementary material for: Phenanthroline-Mediated Photoelectrical Enhancement in Calix[4]arene-Functionalized Titanium-Oxo Clusters
Source: Molecules. 2024 May 30;29(11):2566. doi: 10.3390/molecules29112566 (PMC11173645; doi:10.3390/molecules29112566)
Supplement: Supplementary file 1 [file molecules-29-02566-s001.zip › molecules-3002188-supplementary/Supplementary Files-molecules-3002188-R2/SI-molecules-3002188-R2.pdf]

Supporting Information

Phenanthroline-Mediated Photoelectrical Enhancement in Calix[4]arene-Functionalized Titanium-Oxo Clusters

Jinle Hou *, Chen Huang, Yuxin Liu, Pengfei Fei, Dongxu Zhang, Konggang Qu, Wenwen Zi and Xianqiang Huang *

Shandong Provincial Key Laboratory of Chemical Energy Storage and Novel Cell Technology, School of Chemistry and Chemical Engineering, Liaocheng University, Liaocheng 252000, China; 19861527937@163.com (C.H.); 17861812770@163.com (Y.L.); 18863565739@163.com (P.F.); 15689054231@163.com (D.Z.); qukonggang@lcu.edu.cn (K.Q.); ziwenwen@lcu.edu.cn (W.Z.)

* Correspondence: houjinle@lcu.edu.cn (J.H.); hxq@lcu.edu.cn (X.H.)

Figure S1. The compared PXRD patterns of **Ti2**.

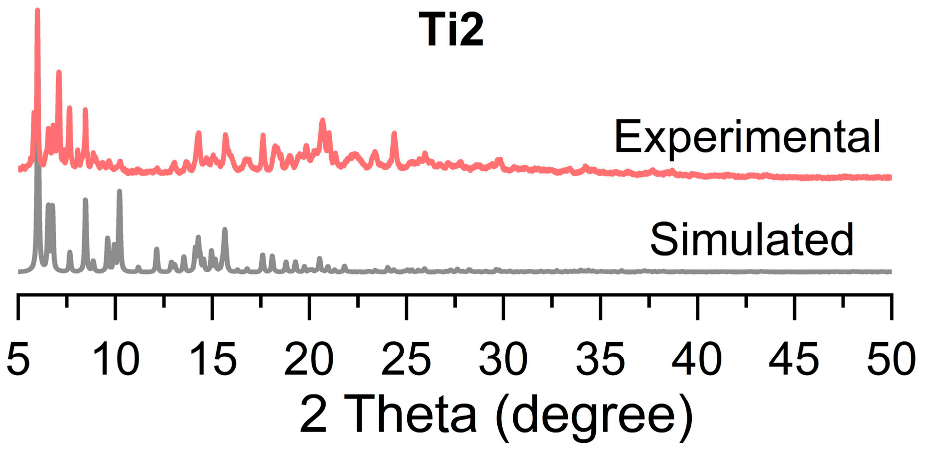

Figure S2. The compared PXRD patterns of **Ti2-Phen**.

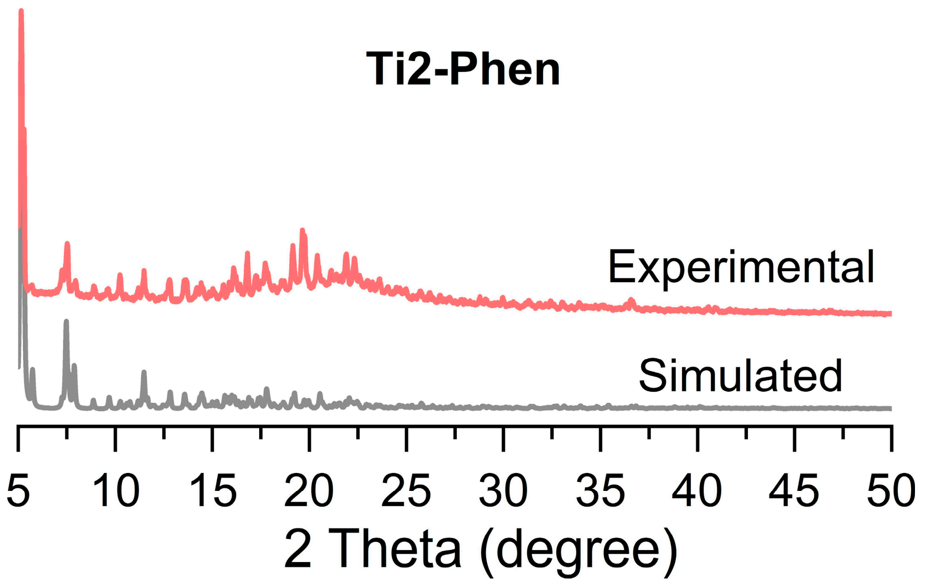

Figure S3. The compared PXRD patterns of **Ti2-BPhen**.

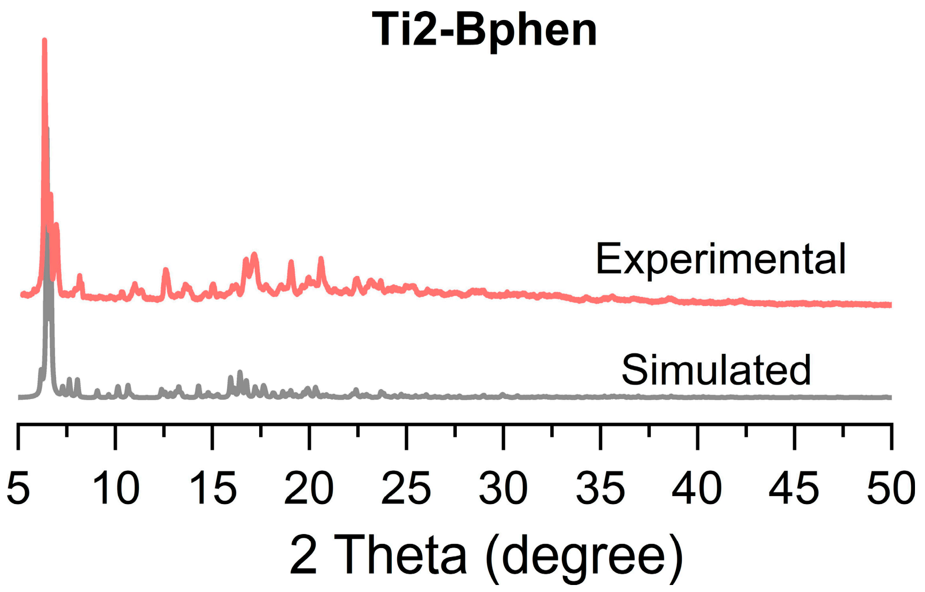

Figure S4. The Fourier transform infrared spectroscopy (FT-IR) of **Ti2**, **Ti2-Phen**, and **Ti2-Bphen**.

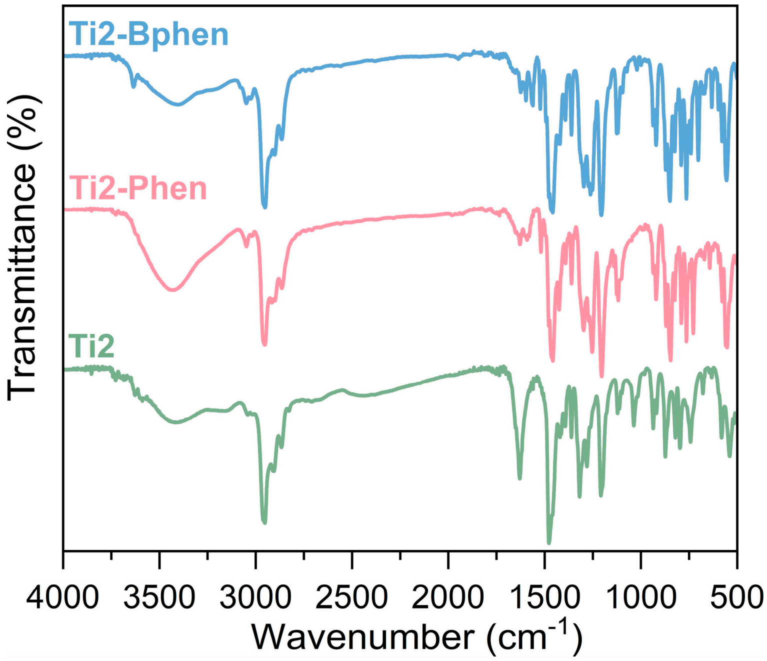

Figure S5. Elemental mapping images of Ti, O, and C of selected area for **Ti2**.

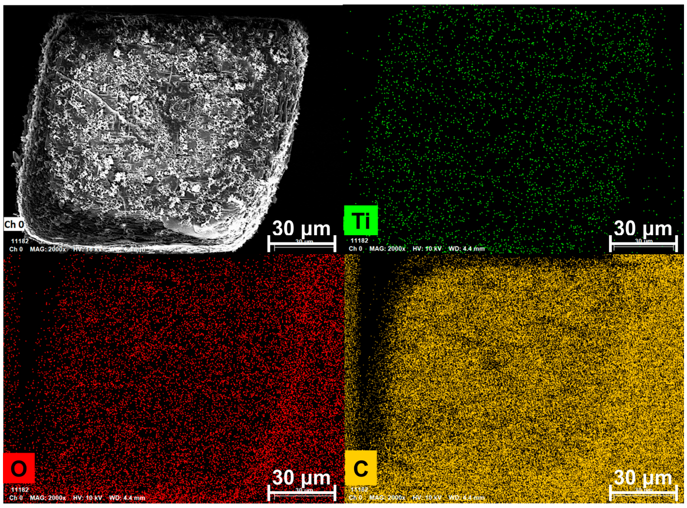

Figure S6. Elemental mapping images of Ti, O, and N of selected area for **Ti2-Phen**.

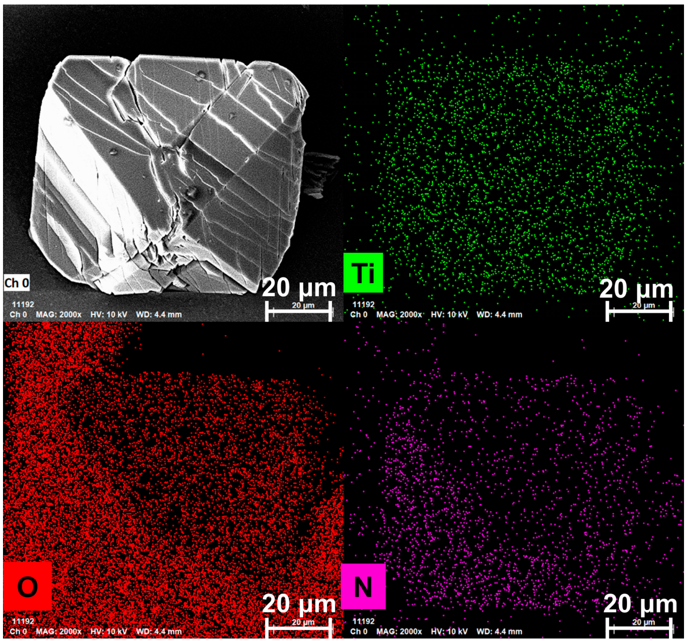

Figure S7. Elemental mapping images of Ti, O, and N of selected area for **Ti2-Bphen**.

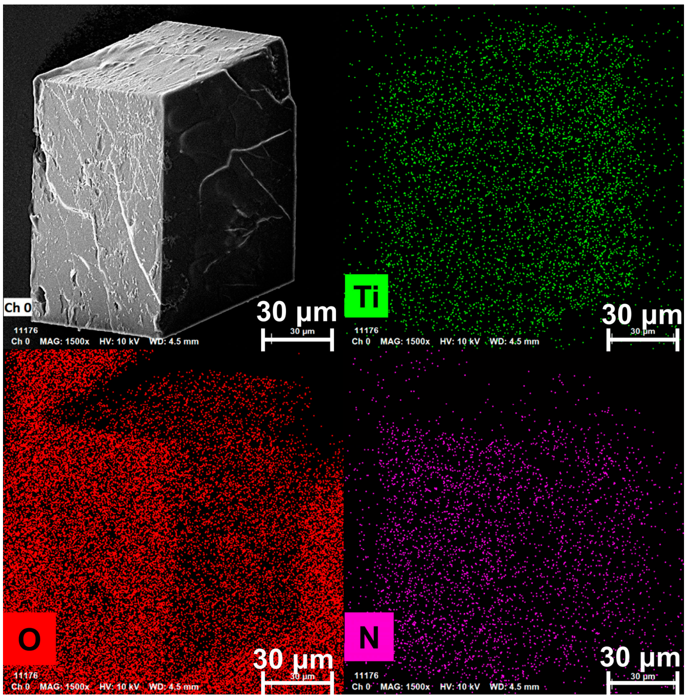

Figure S8. X-ray photoelectron spectroscopy (XPS) spectra of **Ti2**.

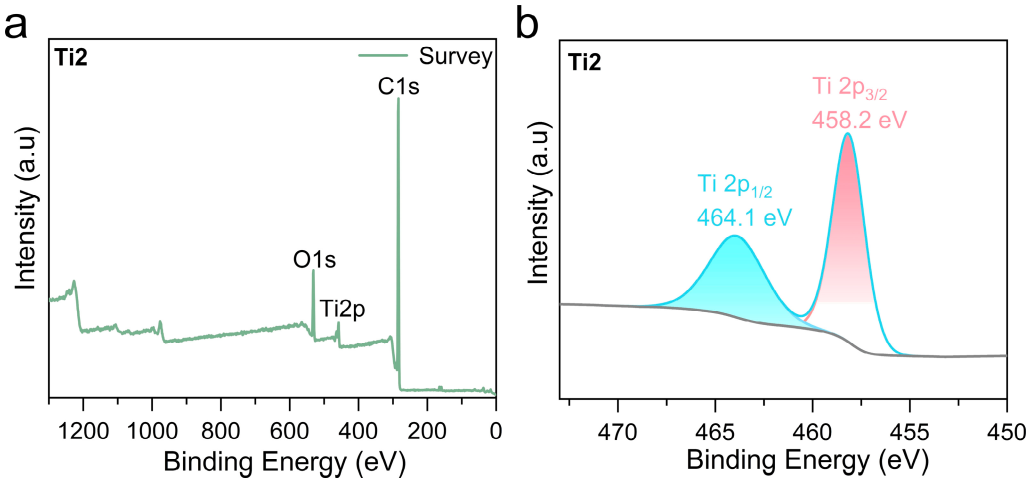

Figure S9. X-ray photoelectron spectroscopy (XPS) spectra of **Ti2-Phen**.

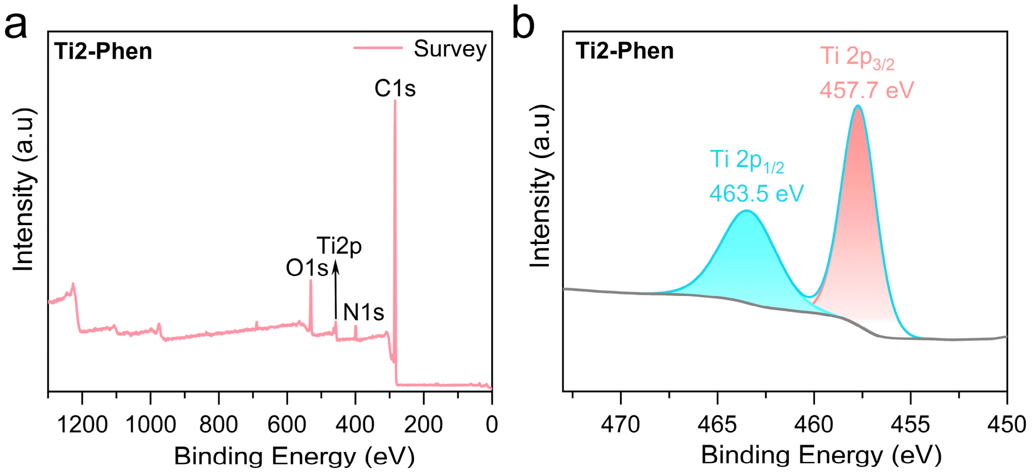

Figure S10. X-ray photoelectron spectroscopy (XPS) spectra of **Ti2-Bphen**.

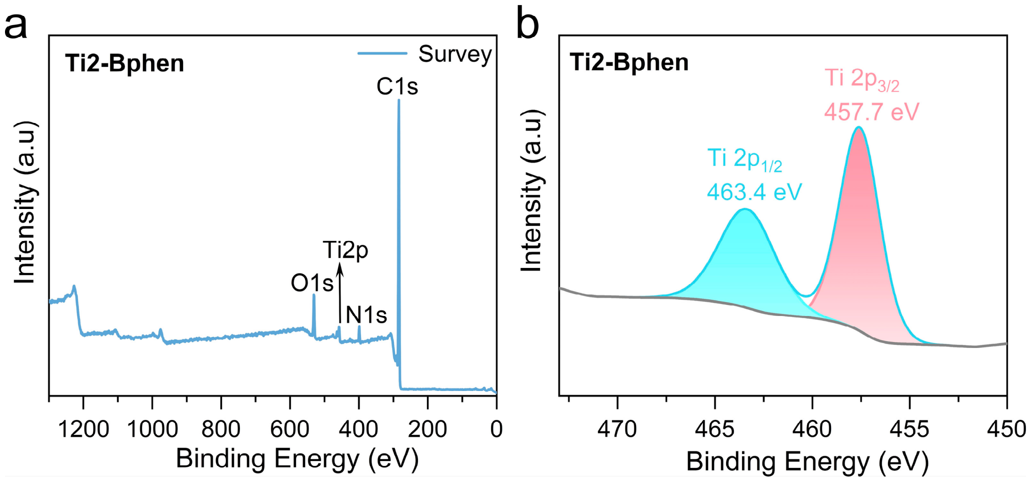

Figure S11. PXRD patterns of **Ti2-Bphen** immersed in water solutions with pH values of 1, 7, and 11 for 24 hours.

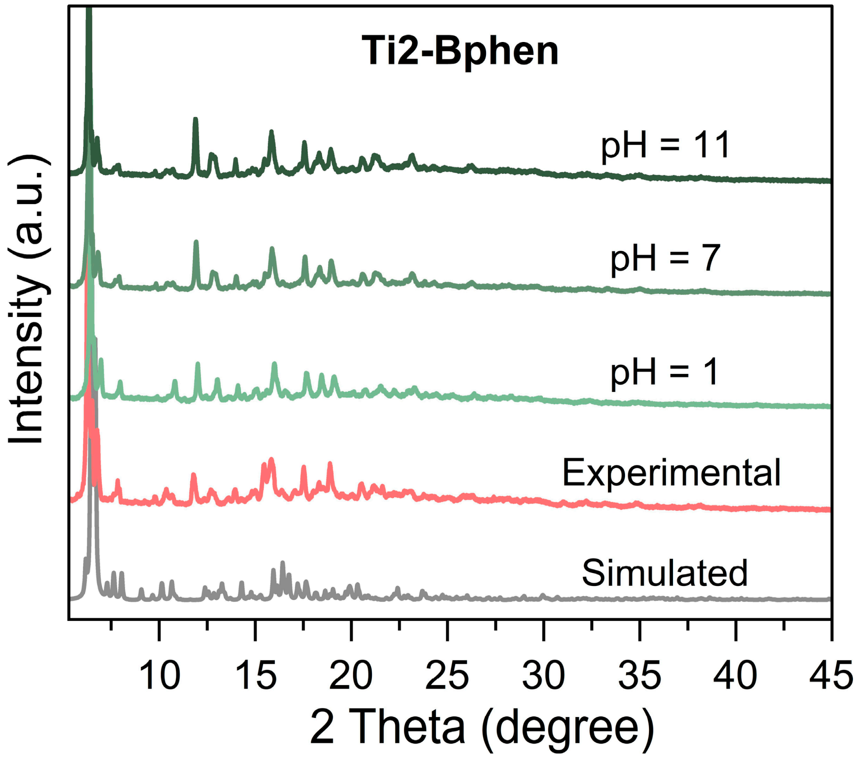

Figure S12. The thermogravimetric analysis (TGA) curves of **Ti2**, **Ti2-Phen**, and **Ti2-Bphen**.

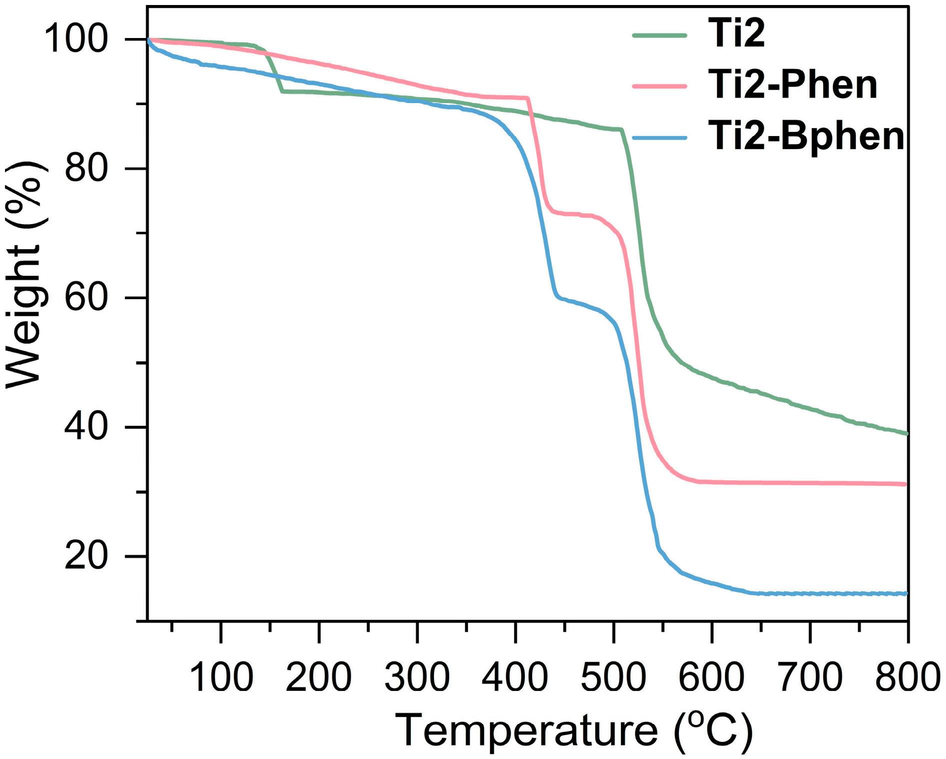

Figure S13. The PXRD patterns of **Ti2-Phen** before and after photocurrent testing.

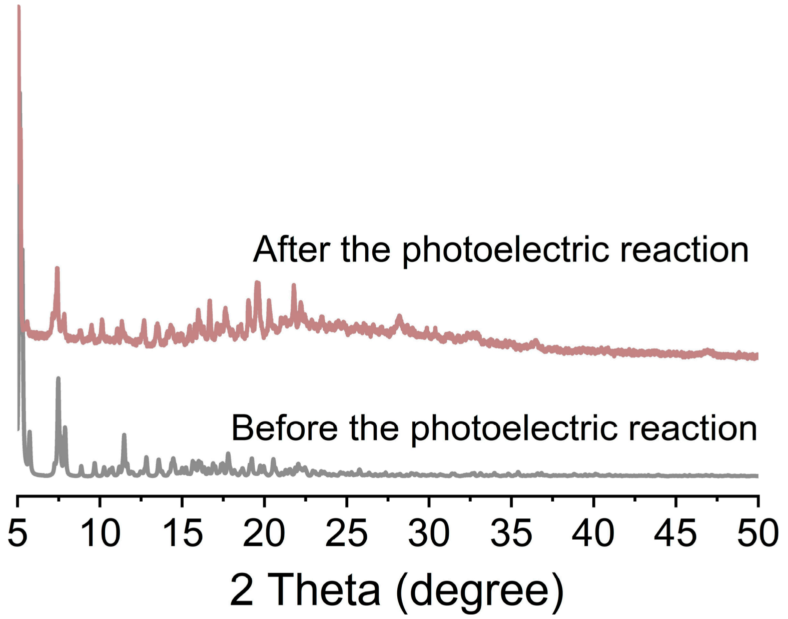

Figure S14. The six cycles of photocurrent testing of **Ti2-Phen**.

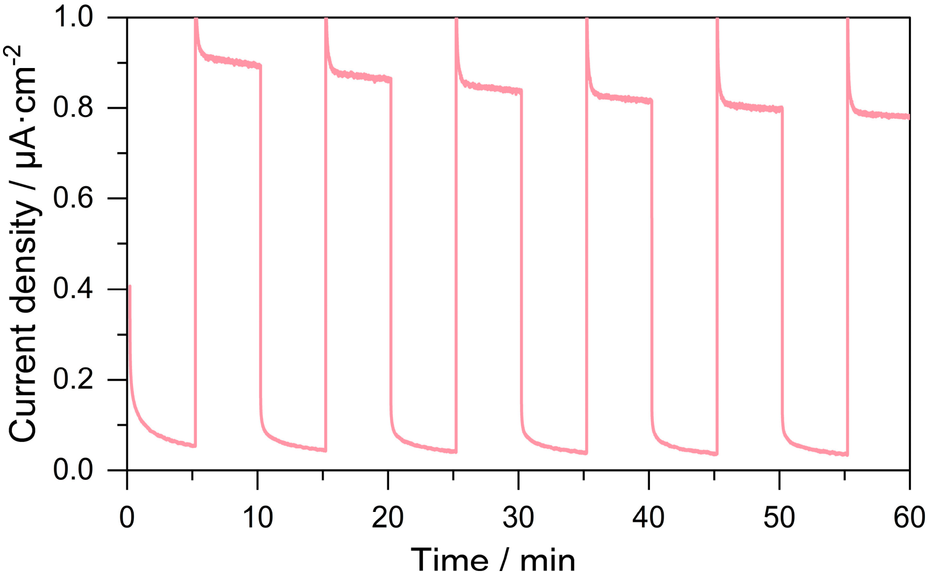

Table S1. Exploration of the synthesis of **Ti2-Phen**.

TBC4A (mmol)	Phen (mmol)	Ti(^{i}PrO)$_4$ (mmol)	Solvent (5 mL)	Temperature (^{o}C)	Result
0.10	0.30	0.66	isopropanol	100	√
0.10	0.30	0.66	isopropanol	80	×
0.10	0.30	0.66	methanol	100	×
0.10	0.30	0.66	ethanol	100	×
0.10	0.30	0.66	acetonitrile	100	×

Table S2. Crystal data collection and structure refinement for **Ti2**, **Ti2-Phen**, and **Ti2-Bphen**.

Compound	Ti2	Ti2-Phen	Ti2-Bphen
Empirical formula	$C_{90}H_{110}O_{10}Ti_2$	$C_{112}H_{120}N_4O_8Ti_2$	$C_{136}H_{136}N_4O_8Ti_2$
Formula weight	1447.57	1745.90	2050.29
Temperature [K]	293.0	173.0	173.0
Crystal system	triclinic	monoclinic	monoclinic
Space group (number)	$P\bar{1}$ (2)	$C2/c$ (15)	$C2/m$ (12)
a [Å]	13.2106(13)	45.092(12)	28.604(7)
b [Å]	14.0049(14)	19.851(6)	21.949(3)
c [Å]	15.2780(15)	30.826(8)	21.140(4)
α [°]	104.353(5)	90	90
β [°]	95.764(3)	130.034(10)	92.837(10)
γ [°]	94.865(2)	90	90
Volume [Å^3]	2706.9(5)	21126(10)	13256(5)
Z	1	4	2
ρ_{calc} [g/cm^3]	0.888	1.117	1.027
μ [mm^{-1}]	0.190	1.718	1.425
F(000)	774	7560	4352
Radiation	MoK_α (λ=0.71073 Å)	CuK_α (λ=1.54178 Å)	CuK_α (λ=1.54178 Å)
2θ range [°]	4.09 to 50.04 (0.84 Å)	5.74 to 134.15 (0.84 Å)	5.08 to 134.32 (0.84 Å)
Index ranges	$-15 \le h \le 15$<br>$-12 \le k \le 16$<br>$-18 \le l \le 11$	$-53 \le h \le 53$<br>$-23 \le k \le 23$<br>$-36 \le l \le 34$	$-34 \le h \le 32$<br>$-26 \le k \le 26$<br>$-24 \le l \le 25$
Reflections collected	13255	85697	61763
Independent reflections	9389<br>R_{int} = 0.0808<br>R_{sigma} = 0.1366	18826<br>R_{int} = 0.0371<br>R_{sigma} = 0.0288	12080<br>R_{int} = 0.0664<br>R_{sigma} = 0.0505
Completeness	98.2 %	99.7 %	98.9 %
Data / Restraints / Parameters	9389/360/566	18826/809/1415	12080/668/871
Goodness-of-fit on F^2	1.137	1.063	1.411
Final R indexes [$I \geq 2\sigma(I)$]	R_1 = 0.1007<br>wR_2 = 0.2511	R_1 = 0.0420<br>wR_2 = 0.1159	R_1 = 0.1097<br>wR_2 = 0.3349
Final R indexes [all data]	R_1 = 0.1671<br>wR_2 = 0.2778	R_1 = 0.0489<br>wR_2 = 0.1211	R_1 = 0.1329<br>wR_2 = 0.3554
Largest peak/hole [e/Å^3]	0.63/-0.53	0.43/-0.43	0.95/-1.41

Table S3. Comparison of crystallographic parameters for **Ti2** and **Ti2-MeOH**.

Compound	**Ti2**	**Ti2-MeOH**
Crystal system	triclinic	monoclinic
Space group	*P* -1	$P\ 2_1/n$
a [Å]	13.2106	19.8135
b [Å]	14.0049	22.9853
c [Å]	15.2780	23.1531
α [°]	104.353	90
β [°]	95.764	102.0212
γ [°]	94.865	90
Volume [$Å^3$]	2706.85	10313.1

Table S4. Comparison of synthesis details for **Ti2** and **Ti2-MeOH**.

Compound	**Ti2**	**Ti2-MeOH**
TBC4A (mmol)	0.10	0.032
$Ti(O^iPr)_4$ (mmol)	0.66	0.33
MeOH (mL)	3	3
DMF (mL)	2	0
reaction temperature (°C)	100	80
reaction time (d)	5	2
