# Supplementary material for: Phenanthroline-Mediated Photoelectrical Enhancement in Calix[4]arene-Functionalized Titanium-Oxo Clusters
Source: Molecules. 2024 May 30;29(11):2566. doi: 10.3390/molecules29112566 (PMC11173645; doi:10.3390/molecules29112566)

checkCIF/PLATON report

Structure factors have been supplied for datablock(s) Ti2-Bphen

THIS REPORT IS FOR GUIDANCE ONLY. IF USED AS PART OF A REVIEW PROCEDURE FOR PUBLICATION, IT SHOULD NOT REPLACE THE EXPERTISE OF AN EXPERIENCED CRYSTALLOGRAPHIC REFEREE.

No syntax errors found. CIF dictionary Interpreting this report

Datablock: Ti2-Bphen

Bond precision:	C-C = 0.0076 A		Wavelength=1.54178
Cell:	a=28.604(7)	b=21.949(3)	c=21.140(4)
	alpha=90	beta=92.837(10)	gamma=90
Temperature:	173 K		

	Calculated	Reported
Volume	13256(4)	13256(5)
Space group	C 2/m	C 1 2/m 1
Hall group	-C 2y	-C 2y
Moiety formula	C136 H136 N4 O8 Ti2 [+ solvent]	2(C136 H136 N4 O8 Ti2)
Sum formula	C136 H136 N4 O8 Ti2 [+ solvent]	C272 H272 N8 O16 Ti4
Mr	2050.23	4100.56
Dx,g cm-3	1.027	1.027
Z	4	2
Mu (mm-1)	1.425	1.425
F000	4352.0	4352.0
F000'	4365.13	
h,k,lmax	34,26,25	34,26,25
Nref	12213	12080
Tmin,Tmax	0.831,0.867	0.615,0.753
Tmin'	0.831	

Correction method= # Reported T Limits: Tmin=0.615 Tmax=0.753
AbsCorr = MULTI-SCAN

Data completeness= 0.989 Theta(max)= 67.160

R(reflections)= 0.1097(8670) wR2(reflections)= 0.3554(12080)

S = 1.411 Npar= 871

The following ALERTS were generated. Each ALERT has the format
test-name_ALERT_alert-type_alert-level.
Click on the hyperlinks for more details of the test.

Alert level B

```
PLAT084_ALERT_3_B High wR2 Value (i.e. > 0.25) ..................       0.36 Report
```

Alert level C

```
PLAT082_ALERT_2_C High R1 Value .................................       0.11 Report
PLAT220_ALERT_2_C NonSolvent   Resd 1  C   Ueq(max)/Ueq(min) Range       3.3 Ratio
PLAT341_ALERT_3_C Low Bond Precision on  C-C Bonds ...............   0.00757 Ang.
PLAT911_ALERT_3_C Missing FCF Refl Between Thmin & STh/L=    0.598       132 Report
                    1  9  0,   2  0  0,   4 26  0,  22 20  0,  34  0  0,  34  2  0,
                  -34  0  1, -34  2  1, -22 20  1,  -5  1  1,  -4  0  1,  -4 26  1,
                   -1  1  1,   2  0  1,   4 26  1,   6  4  1,  22 20  1,  34  0  1,
                  -34  0  2, -34  2  2, -22 20  2,  -6  2  2,   0  0  2,   5  5  2,
                    7  5  2,   8  2  2, -34  2  3, -11  7  3,  -9 13  3,  -5  3  3,
                   -2 26  3,   0  0  3,   0 26  3,   2  0  3,   9 25  3,  23 19  3,
                   29 13  3, -23 19  4, -18 22  4,  -9  3  4,  -9 25  4,   0  0  4,
                    2  0  4,   2  6  4,   3  5  4, -15 23  5, -12 24  5,   6  0  5,
                    7 25  5, -25 17  6, -19 21  6,  -6  4  6,  30 10  6, -33  3  7,
                  -10 24  7,  -4  0  7,   3 25  7,  29 11  7, -17 21  8,   8 24  8,
                   27 13  8, -32  4  9, -22 18  9, -17 21  9,  -6  0  9,   3  1  9,
                   29  9  9, -32  0 10, -27 13 10, -25 15 10, -24 16 10, -18 20 10,
                   -4  0 10,  -2  0 10,   0  0 10,   1  1 10,   2  0 10,   2 24 10,
                  -19 19 11,  23 15 11,  24 14 11,  18 18 12, -30  0 13, -30  2 13,
                   22 14 13,  23 13 13, -29  3 14, -13 19 15,  -3 21 15,   1 21 15,
                  -27  3 16,  25  3 16,   2  0 17,  11 17 17, -25  1 18,  -6 18 18,
PLAT918_ALERT_3_C Reflection(s) with I(obs) much Smaller I(calc) .         2 Check
PLAT972_ALERT_2_C Check Calcd Resid. Dens.  0.11Ang From C42           -1.70 eA-3
PLAT975_ALERT_2_C Check Calcd Resid. Dens.  0.93Ang From C39     .      0.65 eA-3
PLAT977_ALERT_2_C Check Negative Difference Density on H43C      .     -0.44 eA-3
```

Alert level G

```
PLAT002_ALERT_2_G Number of Distance or Angle Restraints on AtSite        25 Note
PLAT003_ALERT_2_G Number of Uiso or Uij Restrained non-H Atoms ...        69 Report
PLAT042_ALERT_1_G Calc. and Reported MoietyFormula Strings  Differ     Please Check
              Calc: C136 H136 N4 O8 Ti2
              Rep.: 2(C136 H136 N4 O8 Ti2)
PLAT045_ALERT_1_G Calculated and Reported Z Differ by a Factor ...         2 Check
PLAT072_ALERT_2_G SHELXL First  Parameter in WGHT  Unusually Large      0.20 Report
PLAT172_ALERT_4_G The CIF-Embedded .res File Contains DFIX Records        18 Report
PLAT178_ALERT_4_G The CIF-Embedded .res File Contains SIMU Records         8 Report
PLAT188_ALERT_3_G A Non-default SIMU Restraint Value has been used    0.0020 Report
PLAT188_ALERT_3_G A Non-default SIMU Restraint Value has been used    0.0020 Report
PLAT188_ALERT_3_G A Non-default SIMU Restraint Value has been used    0.0020 Report
PLAT188_ALERT_3_G A Non-default SIMU Restraint Value has been used    0.0020 Report
PLAT188_ALERT_3_G A Non-default SIMU Restraint Value has been used    0.0020 Report
```

```
PLAT188_ALERT_3_G A Non-default SIMU Restraint Value has been used     0.0020 Report
PLAT188_ALERT_3_G A Non-default SIMU Restraint Value has been used     0.0020 Report
PLAT188_ALERT_3_G A Non-default SIMU Restraint Value has been used     0.0040 Report
PLAT300_ALERT_4_G Atom Site Occupancy of C5        Constrained at        0.5 Check
PLAT300_ALERT_4_G Atom Site Occupancy of C22       Constrained at        0.5 Check
PLAT300_ALERT_4_G Atom Site Occupancy of C40       Constrained at        0.5 Check
PLAT300_ALERT_4_G Atom Site Occupancy of C47       Constrained at        0.5 Check
PLAT300_ALERT_4_G Atom Site Occupancy of C49       Constrained at        0.5 Check
PLAT300_ALERT_4_G Atom Site Occupancy of H5A       Constrained at        0.5 Check
PLAT300_ALERT_4_G Atom Site Occupancy of H5B       Constrained at        0.5 Check
PLAT300_ALERT_4_G Atom Site Occupancy of H5C       Constrained at        0.5 Check
PLAT300_ALERT_4_G Atom Site Occupancy of H22A      Constrained at        0.5 Check
PLAT300_ALERT_4_G Atom Site Occupancy of H22B      Constrained at        0.5 Check
PLAT300_ALERT_4_G Atom Site Occupancy of H22C      Constrained at        0.5 Check
PLAT300_ALERT_4_G Atom Site Occupancy of H39A      Constrained at        0.5 Check
PLAT300_ALERT_4_G Atom Site Occupancy of H39B      Constrained at        0.5 Check
PLAT300_ALERT_4_G Atom Site Occupancy of H39C      Constrained at        0.5 Check
PLAT300_ALERT_4_G Atom Site Occupancy of H40A      Constrained at        0.5 Check
PLAT300_ALERT_4_G Atom Site Occupancy of H40B      Constrained at        0.5 Check
PLAT300_ALERT_4_G Atom Site Occupancy of H40C      Constrained at        0.5 Check
PLAT300_ALERT_4_G Atom Site Occupancy of H47A      Constrained at        0.5 Check
PLAT300_ALERT_4_G Atom Site Occupancy of H47B      Constrained at        0.5 Check
PLAT300_ALERT_4_G Atom Site Occupancy of H47C      Constrained at        0.5 Check
PLAT300_ALERT_4_G Atom Site Occupancy of H49A      Constrained at        0.5 Check
PLAT300_ALERT_4_G Atom Site Occupancy of H49B      Constrained at        0.5 Check
PLAT300_ALERT_4_G Atom Site Occupancy of H49C      Constrained at        0.5 Check
PLAT300_ALERT_4_G Atom Site Occupancy of C28       Constrained at        0.5 Check
PLAT300_ALERT_4_G Atom Site Occupancy of C41       Constrained at        0.5 Check
PLAT300_ALERT_4_G Atom Site Occupancy of C43       Constrained at        0.5 Check
PLAT300_ALERT_4_G Atom Site Occupancy of C48       Constrained at        0.5 Check
PLAT300_ALERT_4_G Atom Site Occupancy of H28A      Constrained at        0.5 Check
PLAT300_ALERT_4_G Atom Site Occupancy of H28B      Constrained at        0.5 Check
PLAT300_ALERT_4_G Atom Site Occupancy of H28C      Constrained at        0.5 Check
PLAT300_ALERT_4_G Atom Site Occupancy of H31A      Constrained at        0.5 Check
PLAT300_ALERT_4_G Atom Site Occupancy of H31B      Constrained at        0.5 Check
PLAT300_ALERT_4_G Atom Site Occupancy of H31C      Constrained at        0.5 Check
PLAT300_ALERT_4_G Atom Site Occupancy of H41A      Constrained at        0.5 Check
PLAT300_ALERT_4_G Atom Site Occupancy of H41B      Constrained at        0.5 Check
PLAT300_ALERT_4_G Atom Site Occupancy of H41C      Constrained at        0.5 Check
PLAT300_ALERT_4_G Atom Site Occupancy of H42A      Constrained at        0.5 Check
PLAT300_ALERT_4_G Atom Site Occupancy of H42B      Constrained at        0.5 Check
PLAT300_ALERT_4_G Atom Site Occupancy of H42C      Constrained at        0.5 Check
PLAT300_ALERT_4_G Atom Site Occupancy of H43A      Constrained at        0.5 Check
PLAT300_ALERT_4_G Atom Site Occupancy of H43B      Constrained at        0.5 Check
PLAT300_ALERT_4_G Atom Site Occupancy of H43C      Constrained at        0.5 Check
PLAT300_ALERT_4_G Atom Site Occupancy of H48A      Constrained at        0.5 Check
PLAT300_ALERT_4_G Atom Site Occupancy of H48B      Constrained at        0.5 Check
PLAT300_ALERT_4_G Atom Site Occupancy of H48C      Constrained at        0.5 Check
PLAT301_ALERT_3_G Main Residue  Disorder .............(Resd    1)        31% Note
PLAT302_ALERT_4_G Anion/Solvent/Minor-Residue Disorder (Resd    2)        21% Note
PLAT333_ALERT_2_G Large Aver C6-Ring C-C Dist C012     -C012_f   .       1.43 Ang.
PLAT367_ALERT_2_G Long?  C(sp?)-C(sp?) Bond  C3       - C4       .       1.54 Ang.
PLAT367_ALERT_2_G Long?  C(sp?)-C(sp?) Bond  C21      - C39      .       1.54 Ang.
PLAT367_ALERT_2_G Long?  C(sp?)-C(sp?) Bond  C31      - C01U     .       1.55 Ang.
PLAT367_ALERT_2_G Long?  C(sp?)-C(sp?) Bond  C32      - C42      .       1.58 Ang.
PLAT412_ALERT_2_G Short Intra XH3 .. XHn     H2       ..H5A      .       1.99 Ang.
                                                     x,y,z  =      1_555 Check
```

```
PLAT412_ALERT_2_G Short Intra XH3 .. XHn     H10      ..H46A     .       1.88 Ang.
                                                   x,y,z  =     1_555 Check
PLAT412_ALERT_2_G Short Intra XH3 .. XHn     H11      ..H15C     .       2.10 Ang.
                                                   x,y,z  =     1_555 Check
PLAT412_ALERT_2_G Short Intra XH3 .. XHn     H11      ..H45B     .       2.08 Ang.
                                                   x,y,z  =     1_555 Check
PLAT412_ALERT_2_G Short Intra XH3 .. XHn     H19      ..H49C     .       1.92 Ang.
                                                   x,y,z  =     1_555 Check
PLAT412_ALERT_2_G Short Intra XH3 .. XHn     H24      ..H41B     .       1.92 Ang.
                                                   x,y,z  =     1_555 Check
PLAT412_ALERT_2_G Short Intra XH3 .. XHn     H24      ..H43B     .       1.72 Ang.
                                                   x,y,z  =     1_555 Check
PLAT412_ALERT_2_G Short Intra XH3 .. XHn     H25      ..H48A     .       2.03 Ang.
                                                  x,-y,z  =     6_555 Check
PLAT606_ALERT_4_G Solvent Accessible VOID(S) in Structure ........          ! Info
PLAT720_ALERT_4_G Number of Unusual/Non-Standard Labels ..........         61 Note
              Ti01    Ti02    O003    O004    O005    O006    O007    O008
              C00A    C00B    C00C    C00D    C00G    C00H    C00I    H00I
              C00K    C00Q    C00U    C00X    C00Y    C00Z    C010    H01A
              H01B    C011    C012    C013    H01C    H01D    C014    C01B
              C01T    H01T    C020    H020    C01Q    H01Q    C01S    H01S
              C01M    H01M    C01D    H01F    C01E    H01E    C01F    C01G
              C01I    H01I    C01J    C01N    H01G    H01H    H01J    C01P
              H01K    H01L    H01N    C01U    C01V
PLAT779_ALERT_4_G Suspect or Irrelevant (Bond) Angle(s) in CIF ...      22.30 Deg.
              C22  -C21  -C49     6_565   1_555   6_565 ......     #  160 Check
PLAT779_ALERT_4_G Suspect or Irrelevant (Bond) Angle(s) in CIF ...      22.70 Deg.
              C28  -C01U -C48     6_555   1_555   6_555 ......     #  254 Check
PLAT779_ALERT_4_G Suspect or Irrelevant (Bond) Angle(s) in CIF ...      19.70 Deg.
              C41  -C32  -C43     6_555   1_555   6_555 ......     #  326 Check
PLAT789_ALERT_4_G Atoms with Negative _atom_site_disorder_group  #         21 Check
PLAT822_ALERT_4_G CIF-embedded .res Contains Negative PART Numbers          4 Check
PLAT860_ALERT_3_G Number of Least-Squares Restraints ............         668 Note
PLAT868_ALERT_4_G ALERTS Due to the Use of _smtbx_masks Suppressed          ! Info
PLAT883_ALERT_1_G No Info/Value for _atom_sites_solution_primary .     Please Do !
PLAT909_ALERT_3_G Percentage of I>2sig(I) Data at Theta(Max) Still        42% Note
PLAT910_ALERT_3_G Missing # of FCF Reflection(s) Below Theta(Min).          1 Note
                0  0  1,
PLAT913_ALERT_3_G Missing # of Very Strong Reflections in FCF ....          1 Note
               -1  1  1,
PLAT933_ALERT_2_G Number of HKL-OMIT Records in Embedded .res File         16 Note
               -4  0  1,   7  5  2,   2  6  4,  -5  3  3,   5  5  2, -11  7  3,
               -4  0  7,   6  4  1,  -6  4  6,  -5  1  1,   8  2  2,   3  5  4,
                1  9  0,  -9 13  3,  -9  3  4,  -6  2  2,
PLAT969_ALERT_5_G The 'Henn et al.' R-Factor-gap value ..........       6.50 Note
              Predicted wR2: Based on SigI**2  5.47 or SHELX Weight 26.16
PLAT978_ALERT_2_G Number C-C Bonds with Positive Residual Density.          1 Info
```

---

   0 **ALERT level A** = Most likely a serious problem - resolve or explain
   1 **ALERT level B** = A potentially serious problem, consider carefully
   8 **ALERT level C** = Check. Ensure it is not caused by an omission or oversight
  91 **ALERT level G** = General information/check it is not something unexpected

   3 ALERT type 1 CIF construction/syntax error, inconsistent or missing data

```
23 ALERT type 2 Indicator that the structure model may be wrong or deficient
17 ALERT type 3 Indicator that the structure quality may be low
56 ALERT type 4 Improvement, methodology, query or suggestion
 1 ALERT type 5 Informative message, check
```

It is advisable to attempt to resolve as many as possible of the alerts in all categories. Often the minor alerts point to easily fixed oversights, errors and omissions in your CIF or refinement strategy, so attention to these fine details can be worthwhile. In order to resolve some of the more serious problems it may be necessary to carry out additional measurements or structure refinements. However, the purpose of your study may justify the reported deviations and the more serious of these should normally be commented upon in the discussion or experimental section of a paper or in the "special_details" fields of the CIF. checkCIF was carefully designed to identify outliers and unusual parameters, but every test has its limitations and alerts that are not important in a particular case may appear. Conversely, the absence of alerts does not guarantee there are no aspects of the results needing attention. It is up to the individual to critically assess their own results and, if necessary, seek expert advice.

Publication of your CIF in IUCr journals

A basic structural check has been run on your CIF. These basic checks will be run on all CIFs submitted for publication in IUCr journals (*Acta Crystallographica*, *Journal of Applied Crystallography*, *Journal of Synchrotron Radiation*); however, if you intend to submit to *Acta Crystallographica Section C* or *E* or *IUCrData*, you should make sure that full publication checks are run on the final version of your CIF prior to submission.

Publication of your CIF in other journals

Please refer to the *Notes for Authors* of the relevant journal for any special instructions relating to CIF submission.

PLATON version of 06/01/2024; check.def file version of 05/01/2024

Datablock Ti2-Bphen - ellipsoid plot

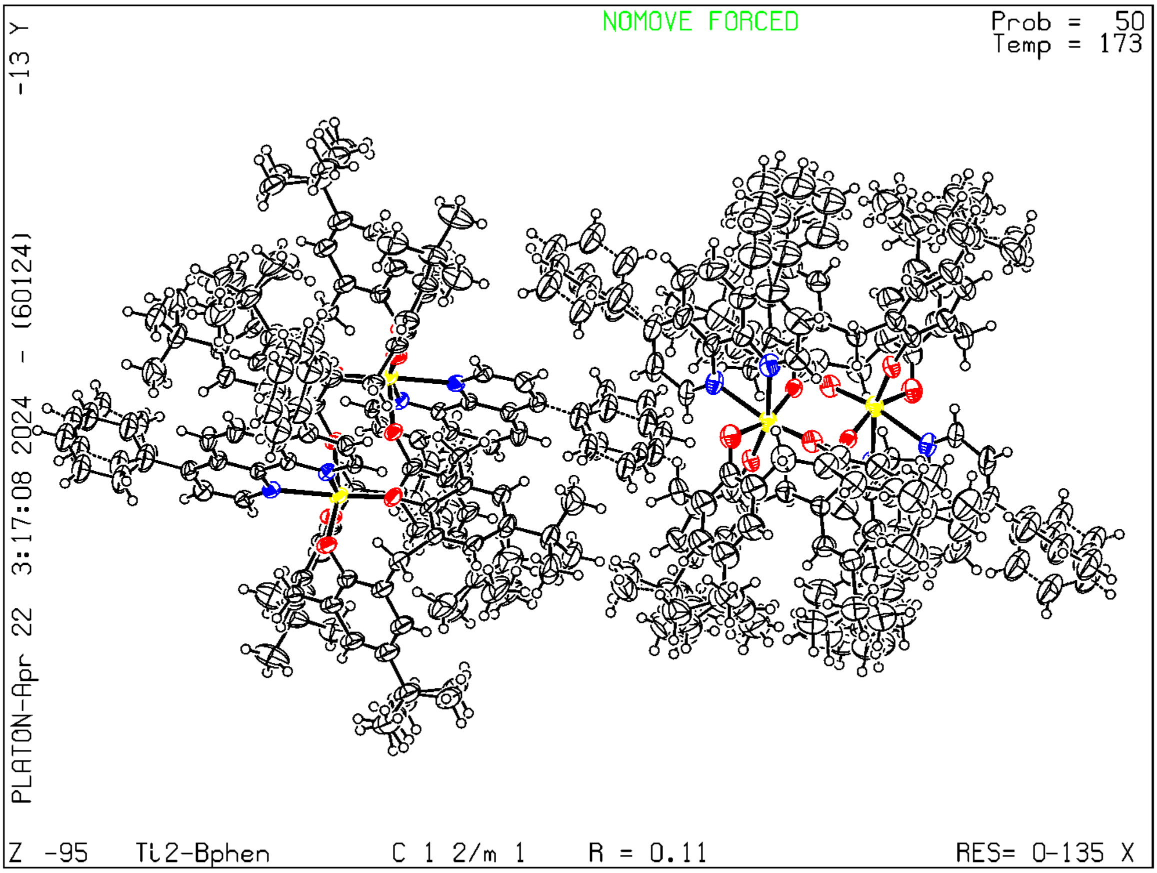

Supplement: Supplementary file 1 [file molecules-29-02566-s001.zip › molecules-3002188-supplementary/Supplementary Files-molecules-3002188-R2/Ti2-Bphen-checkcif.pdf]
