# Supplementary material for: Phenanthroline-Mediated Photoelectrical Enhancement in Calix[4]arene-Functionalized Titanium-Oxo Clusters
Source: Molecules. 2024 May 30;29(11):2566. doi: 10.3390/molecules29112566 (PMC11173645; doi:10.3390/molecules29112566)

checkCIF/PLATON report

Structure factors have been supplied for datablock(s) Ti2-Phen

THIS REPORT IS FOR GUIDANCE ONLY. IF USED AS PART OF A REVIEW PROCEDURE FOR PUBLICATION, IT SHOULD NOT REPLACE THE EXPERTISE OF AN EXPERIENCED CRYSTALLOGRAPHIC REFEREE.

No syntax errors found. CIF dictionary Interpreting this report

Datablock: Ti2-Phen

Bond precision: C-C = 0.0033 A Wavelength=1.54178

Cell:	a=45.092(12)	b=19.851(6)	c=30.826(8)
	alpha=90	beta=130.034(10)	gamma=90
Temperature:	173 K		

	Calculated	Reported
Volume	21127(11)	21126(10)
Space group	C 2/c	C 1 2/c 1
Hall group	-C 2yc	-C 2yc
Moiety formula	2(C112 H120 N4 O8 Ti2), C3 H8 O [+ solvent]	2(C112 H120 N4 O8 Ti2), C3 H8 O
Sum formula	C227 H248 N8 O17 Ti4 [+ solvent]	C227 H248 N8 O17 Ti4
Mr	3551.81	3551.92
Dx,g cm-3	1.117	1.117
Z	4	4
Mu (mm-1)	1.718	1.718
F000	7560.0	7560.0
F000'	7583.35	
h,k,lmax	53,23,36	53,23,36
Nref	18881	18826
Tmin,Tmax	0.806,0.996	0.617,0.754
Tmin'	0.806	

Correction method= # Reported T Limits: Tmin=0.617 Tmax=0.754
AbsCorr = MULTI-SCAN

Data completeness= 0.997 Theta(max)= 67.075

R(reflections)= 0.0420(16242)   wR2(reflections)= 0.1211(18826)

S = 1.063   Npar= 1415

The following ALERTS were generated. Each ALERT has the format
test-name_ALERT_alert-type_alert-level.
Click on the hyperlinks for more details of the test.

Alert level C

```
PLAT220_ALERT_2_C NonSolvent   Resd 1  C   Ueq(max)/Ueq(min) Range        5.5 Ratio
PLAT221_ALERT_2_C Solv./Anion  Resd 2  C   Ueq(max)/Ueq(min) Range        4.8 Ratio
PLAT222_ALERT_3_C NonSolvent Resd 1  H   Uiso(max)/Uiso(min) Range        6.0 Ratio
PLAT223_ALERT_4_C Solv./Anion  Resd 2  H   Ueq(max)/Ueq(min) Range        5.0 Ratio
PLAT242_ALERT_2_C Low    'MainMol' Ueq as Compared to Neighbors of        C90 Check
PLAT242_ALERT_2_C Low    'MainMol' Ueq as Compared to Neighbors of        C49 Check
PLAT242_ALERT_2_C Low    'MainMol' Ueq as Compared to Neighbors of        C51 Check
PLAT242_ALERT_2_C Low    'MainMol' Ueq as Compared to Neighbors of        C76 Check
PLAT790_ALERT_4_C Centre of Gravity not Within Unit Cell: Resd.  #          1 Note
              C112 H120 N4 O8 Ti2
PLAT911_ALERT_3_C Missing FCF Refl Between Thmin & STh/L=    0.597         48 Report
                1 23  0,   3  1  0,   6  2  0, -16  2  1,  -4  4  1,  -3  1  1,
               -1 23  1,   1  1  1,   1 23  1,   3 23  1,   9 23  1,  -4  0  2,
               -1  1  2,   0  0  2,   2  0  2,   3  3  2,   5  1  2,  -5  3  3,
               -2  4  3,   0  2  3,  -8  0  4,  -6  0  4,  -6  4  4,   0  2  4,
               -4  0  6, -12  0  8, -18  4  9, -18  4 10, -13  5 11, -18  4 12,
               12  0 12, -20  0 16, -29 19 19, -28 18 22, -24  2 23, -28 16 25,
              -25  1 25, -26  0 26, -27 15 27, -30  0 28, -28 14 28, -26  0 28,
              -27 13 30, -47  1 34, -46  0 34, -44  0 34, -45  1 35, -42  0 36,
PLAT913_ALERT_3_C Missing # of Very Strong Reflections in FCF ....          8 Note
                1  1  0,  -1  1  1,  -4  0  2,   2  0  2,   3  3  2,  -8  0  4,
               -6  0  4,  -6  4  4,
```

Alert level G

```
PLAT002_ALERT_2_G Number of Distance or Angle Restraints on AtSite         47 Note
PLAT003_ALERT_2_G Number of Uiso or Uij Restrained non-H Atoms ...         91 Report
PLAT007_ALERT_5_G Number of Unrefined Donor-H Atoms .............          1 Report
              H2
PLAT083_ALERT_2_G SHELXL Second Parameter in WGHT  Unusually Large      13.68 Why ?
PLAT128_ALERT_4_G Alternate Setting for Input Space Group      C2/c      I2/a Note
PLAT172_ALERT_4_G The CIF-Embedded .res File Contains DFIX Records         19 Report
PLAT178_ALERT_4_G The CIF-Embedded .res File Contains SIMU Records          8 Report
PLAT188_ALERT_3_G A Non-default SIMU Restraint Value has been used     0.0040 Report
PLAT188_ALERT_3_G A Non-default SIMU Restraint Value has been used     0.0040 Report
PLAT188_ALERT_3_G A Non-default SIMU Restraint Value has been used     0.0040 Report
PLAT188_ALERT_3_G A Non-default SIMU Restraint Value has been used     0.0040 Report
PLAT188_ALERT_3_G A Non-default SIMU Restraint Value has been used     0.0040 Report
PLAT188_ALERT_3_G A Non-default SIMU Restraint Value has been used     0.0040 Report
PLAT188_ALERT_3_G A Non-default SIMU Restraint Value has been used     0.0020 Report
PLAT188_ALERT_3_G A Non-default SIMU Restraint Value has been used     0.0020 Report
PLAT300_ALERT_4_G Atom Site Occupancy of O2         Constrained at        0.5 Check
PLAT300_ALERT_4_G Atom Site Occupancy of C38        Constrained at        0.5 Check
PLAT300_ALERT_4_G Atom Site Occupancy of C100       Constrained at        0.5 Check
PLAT300_ALERT_4_G Atom Site Occupancy of C102       Constrained at        0.5 Check
```

```
PLAT300_ALERT_4_G Atom Site Occupancy of H2          Constrained at        0.5 Check
PLAT300_ALERT_4_G Atom Site Occupancy of H10J        Constrained at        0.5 Check
PLAT300_ALERT_4_G Atom Site Occupancy of H10K        Constrained at        0.5 Check
PLAT300_ALERT_4_G Atom Site Occupancy of H10L        Constrained at        0.5 Check
PLAT300_ALERT_4_G Atom Site Occupancy of H38A        Constrained at        0.5 Check
PLAT300_ALERT_4_G Atom Site Occupancy of H38B        Constrained at        0.5 Check
PLAT300_ALERT_4_G Atom Site Occupancy of H38C        Constrained at        0.5 Check
PLAT300_ALERT_4_G Atom Site Occupancy of H100        Constrained at        0.5 Check
PLAT301_ALERT_3_G Main Residue  Disorder .............(Resd    1)         19% Note
PLAT302_ALERT_4_G Anion/Solvent/Minor-Residue Disorder (Resd    2)         14% Note
PLAT302_ALERT_4_G Anion/Solvent/Minor-Residue Disorder (Resd    3)        100% Note
PLAT412_ALERT_2_G Short Intra XH3 .. XHn     H3AA     ..H103     .        2.14 Ang.
                                                        x,y,z  =     1_555 Check
PLAT412_ALERT_2_G Short Intra XH3 .. XHn     H32      ..H6AB     .        1.96 Ang.
                                                        x,y,z  =     1_555 Check
PLAT412_ALERT_2_G Short Intra XH3 .. XHn     H47      ..H4AB     .        2.07 Ang.
                                                        x,y,z  =     1_555 Check
PLAT412_ALERT_2_G Short Intra XH3 .. XHn     H83      ..H94B     .        2.07 Ang.
                                                        x,y,z  =     1_555 Check
PLAT412_ALERT_2_G Short Intra XH3 .. XHn     H85      ..H12A     .        1.98 Ang.
                                                        x,y,z  =     1_555 Check
PLAT412_ALERT_2_G Short Intra XH3 .. XHn     H93      ..H21C     .        2.11 Ang.
                                                        x,y,z  =     1_555 Check
PLAT412_ALERT_2_G Short Intra XH3 .. XHn     H15B     ..H29      .        2.10 Ang.
                                                        x,y,z  =     1_555 Check
PLAT412_ALERT_2_G Short Intra XH3 .. XHn     H29      ..H15E     .        2.01 Ang.
                                                        x,y,z  =     1_555 Check
PLAT412_ALERT_2_G Short Intra XH3 .. XHn     H36      ..H10G     .        1.98 Ang.
                                                        x,y,z  =     1_555 Check
PLAT412_ALERT_2_G Short Intra XH3 .. XHn     H63      ..H8C      .        2.11 Ang.
                                                        x,y,z  =     1_555 Check
PLAT432_ALERT_2_G Short Inter X...Y Contact  O2       ..C48      .        2.71 Ang.
                                                        x,y,z  =     1_555 Check
PLAT432_ALERT_2_G Short Inter X...Y Contact  O2       ..C107     .        3.01 Ang.
                                             1-x,-1+y,1/2-z  =     2_645 Check
PLAT432_ALERT_2_G Short Inter X...Y Contact  C25      ..C60      .        3.20 Ang.
                                                1-x,y,1/2-z  =     2_655 Check
PLAT605_ALERT_4_G Largest Solvent Accessible VOID in the Structure        167 A**3
PLAT720_ALERT_4_G Number of Unusual/Non-Standard Labels ..........         27 Note
              H9AA    H9AB    H9AC    H7AA    H7AB    H7AC    H8AA    H8AB
              H8AC    H1AA    H1AB    H1AC    H2AA    H2AB    H2AC    H3AA
              H3AB    H3AC    H5AA    H5AB    H5AC    H4AA    H4AB    H4AC
              H6AA    H6AB    H6AC
PLAT773_ALERT_2_G Check long C-C Bond in CIF: C57      --C5A          1.71 Ang.
PLAT789_ALERT_4_G Atoms with Negative _atom_site_disorder_group  #         12 Check
PLAT822_ALERT_4_G CIF-embedded .res Contains Negative PART Numbers          3 Check
PLAT860_ALERT_3_G Number of Least-Squares Restraints ............         809 Note
PLAT868_ALERT_4_G ALERTS Due to the Use of _smtbx_masks Suppressed          ! Info
PLAT883_ALERT_1_G No Info/Value for _atom_sites_solution_primary .     Please Do !
PLAT909_ALERT_3_G Percentage of I>2sig(I) Data at Theta(Max) Still        74% Note
PLAT910_ALERT_3_G Missing # of FCF Reflection(s) Below Theta(Min).          3 Note
                1  1  0,   2  0  0,  -1  1  1,
PLAT933_ALERT_2_G Number of HKL-OMIT Records in Embedded .res File          1 Note
              -26  0 26,
PLAT941_ALERT_3_G Average HKL Measurement Multiplicity ..........         4.6 Low
PLAT969_ALERT_5_G The 'Henn et al.' R-Factor-gap value ..........        3.55 Note
              Predicted wR2: Based on SigI**2  3.41 or SHELX Weight 11.84
```

```
PLAT978_ALERT_2_G Number C-C Bonds with Positive Residual Density.         10 Info
```

```
   0 ALERT level A = Most likely a serious problem - resolve or explain
   0 ALERT level B = A potentially serious problem, consider carefully
  11 ALERT level C = Check. Ensure it is not caused by an omission or oversight
  57 ALERT level G = General information/check it is not something unexpected

   1 ALERT type 1 CIF construction/syntax error, inconsistent or missing data
  25 ALERT type 2 Indicator that the structure model may be wrong or deficient
  16 ALERT type 3 Indicator that the structure quality may be low
  24 ALERT type 4 Improvement, methodology, query or suggestion
   2 ALERT type 5 Informative message, check
```

It is advisable to attempt to resolve as many as possible of the alerts in all categories. Often the minor alerts point to easily fixed oversights, errors and omissions in your CIF or refinement strategy, so attention to these fine details can be worthwhile. In order to resolve some of the more serious problems it may be necessary to carry out additional measurements or structure refinements. However, the purpose of your study may justify the reported deviations and the more serious of these should normally be commented upon in the discussion or experimental section of a paper or in the "special_details" fields of the CIF. checkCIF was carefully designed to identify outliers and unusual parameters, but every test has its limitations and alerts that are not important in a particular case may appear. Conversely, the absence of alerts does not guarantee there are no aspects of the results needing attention. It is up to the individual to critically assess their own results and, if necessary, seek expert advice.

Publication of your CIF in IUCr journals

A basic structural check has been run on your CIF. These basic checks will be run on all CIFs submitted for publication in IUCr journals (*Acta Crystallographica*, *Journal of Applied Crystallography*, *Journal of Synchrotron Radiation*); however, if you intend to submit to *Acta Crystallographica Section C* or *E* or *IUCrData*, you should make sure that full publication checks are run on the final version of your CIF prior to submission.

Publication of your CIF in other journals

Please refer to the *Notes for Authors* of the relevant journal for any special instructions relating to CIF submission.

PLATON version of 06/01/2024; check.def file version of 05/01/2024

Datablock Ti2-Phen - ellipsoid plot

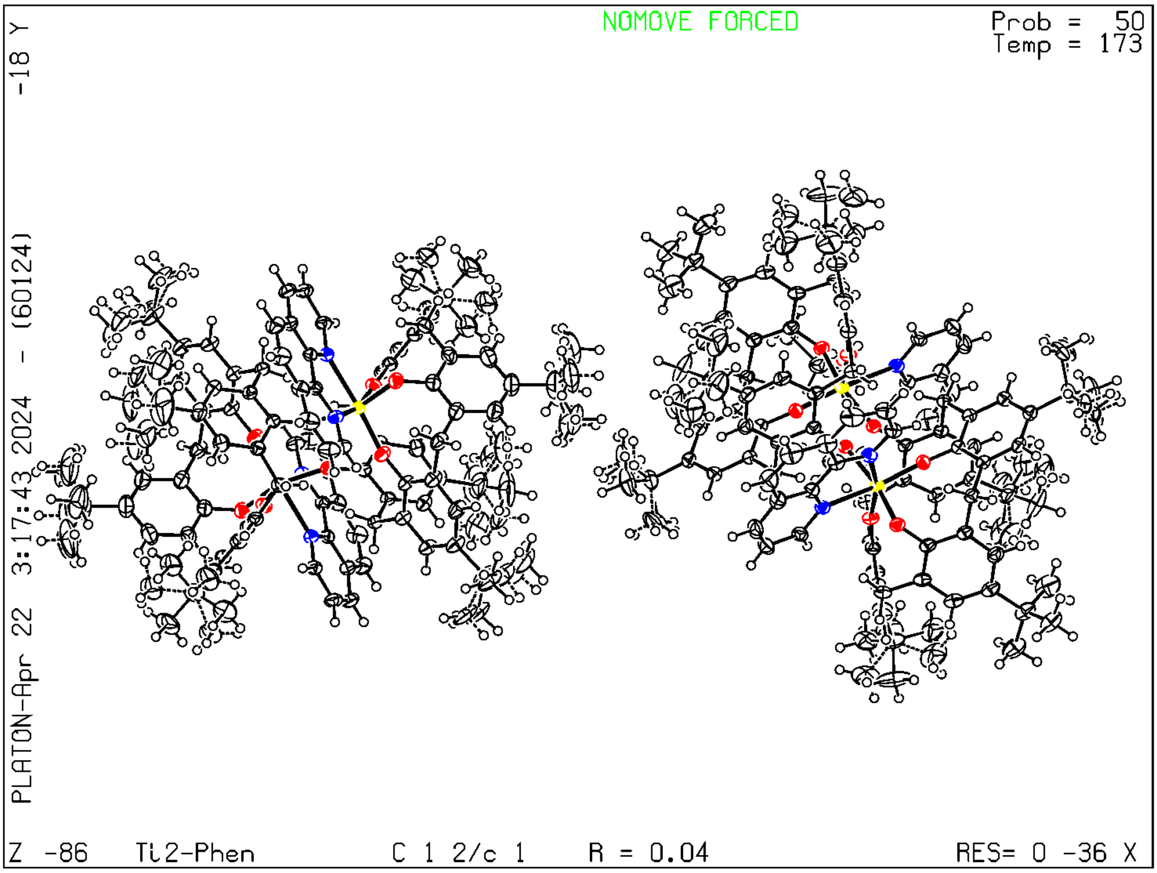

Supplement: Supplementary file 1 [file molecules-29-02566-s001.zip › molecules-3002188-supplementary/Supplementary Files-molecules-3002188-R2/Ti2-Phen-checkcif.pdf]
