# Supplementary material for: Phenanthroline-Mediated Photoelectrical Enhancement in Calix[4]arene-Functionalized Titanium-Oxo Clusters
Source: Molecules. 2024 May 30;29(11):2566. doi: 10.3390/molecules29112566 (PMC11173645; doi:10.3390/molecules29112566)

checkCIF/PLATON report

Structure factors have been supplied for datablock(s) 230429d

THIS REPORT IS FOR GUIDANCE ONLY. IF USED AS PART OF A REVIEW PROCEDURE FOR PUBLICATION, IT SHOULD NOT REPLACE THE EXPERTISE OF AN EXPERIENCED CRYSTALLOGRAPHIC REFEREE.

No syntax errors found.   CIF dictionary   Interpreting this report

Datablock: 230429d

Bond precision: C-C = 0.0084 A   Wavelength=0.71073

Cell:	a=13.2106(13)	b=14.0049(14)	c=15.2780(15)
	alpha=104.353(5)	beta=95.764(3)	gamma=94.865(2)
Temperature:	293 K		

	Calculated	Reported
Volume	2706.9(5)	2706.9(5)
Space group	P -1	P -1
Hall group	-P 1	-P 1
Moiety formula	C90 H112 O10 Ti2 [+ solvent]	C90 H112 O10 Ti2
Sum formula	C90 H112 O10 Ti2 [+ solvent]	C90 H112 O10 Ti2
Mr	1449.54	1449.59
Dx,g cm-3	0.889	0.889
Z	1	1
Mu (mm-1)	0.190	0.190
F000	776.0	776.0
F000'	776.87	
h,k,lmax	15,16,18	15,16,18
Nref	9565	9389
Tmin,Tmax	0.953,0.966	
Tmin'	0.950	

Correction method= Not given

Data completeness= 0.982   Theta(max)= 25.018

R(reflections)= 0.1012(4124)   wR2(reflections)= 0.2798(9389)

S = 1.146   Npar= 569

The following ALERTS were generated. Each ALERT has the format
test-name_ALERT_alert-type_alert-level.
Click on the hyperlinks for more details of the test.

Alert level C

```
PLAT026_ALERT_3_C Ratio Observed / Unique Reflections (too) Low ..        44% Check
PLAT084_ALERT_3_C High wR2 Value (i.e. > 0.25) ...................       0.28 Report
PLAT230_ALERT_2_C Hirshfeld Test Diff for    O5       --C67      .        5.7 s.u.
PLAT341_ALERT_3_C Low Bond Precision on  C-C Bonds ...............    0.00844 Ang.
PLAT905_ALERT_3_C Negative K value in the Analysis of Variance ...     -4.116 Report
PLAT910_ALERT_3_C Missing # of FCF Reflection(s) Below Theta(Min).          5 Note
                1  0  0,   0  1  0,   0 -1  1,  -1  0  1,   0  0  1,
PLAT911_ALERT_3_C Missing FCF Refl Between Thmin & STh/L=    0.595        171 Report
               15  0  0, -13  1  0, -15  2  0,  -2  3  0,  14  4  0,  -2-15  1,
                1 -4  1,   4 -3  1,  -4 -1  1, -11  0  1,  -5  3  1,   2 -4  2,
                1 -3  2,   4 -3  2,  14 -2  2, -14  1  2,   1  1  2,  -5  3  2,
               15 -3  3,  -1 -2  3, -12  0  3,   0  1  3,   3  1  3,   8-14  4,
               -9 -1  4,   0  1  4,   2  1  4,   3 10  4,  -9 -1  5,  -2 -1  5,
                1  0  5,  -9 -1  6,   1  2  6, -13  7  6,  -9 -1  7, -12 -9  8,
              -11 -9  8, -12 -8  8, -11 -8  8, -13 -7  8, -12 -7  8, -13 -6  8,
              -12 -6  8, -13 -5  8, -12 -5  8, -14 -4  8, -13 -4  8, -12 -4  8,
              -14 -3  8, -13 -3  8, -14 -2  8, -13 -2  8, -14 -1  8, -13 -1  8,
              -14  0  8, -13  0  8,   0-16  9,   1-16  9,   2-16  9,   0-15  9,
                1-15  9,  -4-14  9, -10-10  9, -12 -7  9, -13 -6  9, -12 -6  9,
              -13 -5  9, -12 -5  9, -11 -5  9, -13 -4  9, -12 -4  9, -11 -4  9,
              -14 -3  9, -13 -3  9, -12 -3  9, -11 -3  9, -14 -2  9, -13 -2  9,
              -12 -2  9, -14 -1  9, -13 -1  9, -12 -1  9, -14  0  9, -13  0  9,
              -14  1  9, -13  1  9, -12  1  9, -11  1  9, -14  2  9, -13  2  9,
              -13  3  9, -13  4  9, -13  5  9, -12  6  9, -12  7  9,   1-15 10,
PLAT934_ALERT_3_C Number of (Iobs-Icalc)/Sigma(W) > 10 Outliers ..          1 Check
                0  0  2,
```

Alert level G

```
PLAT002_ALERT_2_G Number of Distance or Angle Restraints on AtSite         23 Note
PLAT003_ALERT_2_G Number of Uiso or Uij Restrained non-H Atoms ...         43 Report
PLAT172_ALERT_4_G The CIF-Embedded .res File Contains DFIX Records         22 Report
PLAT178_ALERT_4_G The CIF-Embedded .res File Contains SIMU Records          4 Report
PLAT188_ALERT_3_G A Non-default SIMU Restraint Value has been used     0.0020 Report
PLAT188_ALERT_3_G A Non-default SIMU Restraint Value has been used     0.0020 Report
PLAT188_ALERT_3_G A Non-default SIMU Restraint Value has been used     0.0020 Report
PLAT188_ALERT_3_G A Non-default SIMU Restraint Value has been used     0.0020 Report
PLAT199_ALERT_1_G Reported _cell_measurement_temperature ..... (K)        293 Check
PLAT200_ALERT_1_G Reported   _diffrn_ambient_temperature ..... (K)        293 Check
PLAT301_ALERT_3_G Main Residue  Disorder ..............(Resd    1)        18% Note
PLAT303_ALERT_2_G Full Occupancy Atom H1        with # Connections       2.00 Check
PLAT412_ALERT_2_G Short Intra XH3 .. XHn     H21       ..H2B      .      2.09 Ang.
                                                       x,y,z  =     1_555 Check
PLAT412_ALERT_2_G Short Intra XH3 .. XHn     H23       ..H5B      .      2.06 Ang.
                                                       x,y,z  =     1_555 Check
PLAT412_ALERT_2_G Short Intra XH3 .. XHn     H26       ..H50C     .      2.02 Ang.
                                                       x,y,z  =     1_555 Check
PLAT412_ALERT_2_G Short Intra XH3 .. XHn     H30       ..H52C     .      2.12 Ang.
                                                       x,y,z  =     1_555 Check
PLAT412_ALERT_2_G Short Intra XH3 .. XHn     H35       ..H46A     .      2.13 Ang.
```

```
                                                      x,y,z  =      1_555 Check
PLAT606_ALERT_4_G Solvent Accessible VOID(S) in Structure ........         ! Info
PLAT794_ALERT_5_G Tentative Bond Valency for Ti1       (IV)      .       4.28 Info
PLAT860_ALERT_3_G Number of Least-Squares Restraints ............         361 Note
PLAT868_ALERT_4_G ALERTS Due to the Use of _smtbx_masks Suppressed         ! Info
PLAT883_ALERT_1_G No Info/Value for _atom_sites_solution_primary .     Please Do !
PLAT913_ALERT_3_G Missing # of Very Strong Reflections in FCF ....          1 Note
                0  0  1,
PLAT933_ALERT_2_G Number of HKL-OMIT Records in Embedded .res File         18 Note
                4 -3  2,  -2 -1  5,  -2  3  0,  -1 -2  3,  -4 -1  1,   1 -4  1,
                3  1  3,   1 -3  2,   2 -4  2,   0  1  3,   0  1  4,   4 -3  1,
                2  1  4,  -5  3  2,   1  2  6,   1  1  2,  -5  3  1,   1  0  5,
PLAT941_ALERT_3_G Average HKL Measurement Multiplicity ..........        1.4 Low
PLAT967_ALERT_5_G Note: Two-Theta Cutoff Value in Embedded .res ..       50.0 Degree
PLAT969_ALERT_5_G The 'Henn et al.' R-Factor-gap value ..........       2.79 Note
              Predicted wR2: Based on SigI**2 10.02 or SHELX Weight 25.19
PLAT978_ALERT_2_G Number C-C Bonds with Positive Residual Density.          2 Info
```

---

   0 **ALERT level A** = Most likely a serious problem - resolve or explain
   0 **ALERT level B** = A potentially serious problem, consider carefully
   8 **ALERT level C** = Check. Ensure it is not caused by an omission or oversight
  28 **ALERT level G** = General information/check it is not something unexpected

   3 ALERT type 1 CIF construction/syntax error, inconsistent or missing data
  11 ALERT type 2 Indicator that the structure model may be wrong or deficient
  15 ALERT type 3 Indicator that the structure quality may be low
   4 ALERT type 4 Improvement, methodology, query or suggestion
   3 ALERT type 5 Informative message, check

It is advisable to attempt to resolve as many as possible of the alerts in all categories. Often the minor alerts point to easily fixed oversights, errors and omissions in your CIF or refinement strategy, so attention to these fine details can be worthwhile. In order to resolve some of the more serious problems it may be necessary to carry out additional measurements or structure refinements. However, the purpose of your study may justify the reported deviations and the more serious of these should normally be commented upon in the discussion or experimental section of a paper or in the "special_details" fields of the CIF. checkCIF was carefully designed to identify outliers and unusual parameters, but every test has its limitations and alerts that are not important in a particular case may appear. Conversely, the absence of alerts does not guarantee there are no aspects of the results needing attention. It is up to the individual to critically assess their own results and, if necessary, seek expert advice.

Publication of your CIF in IUCr journals

A basic structural check has been run on your CIF. These basic checks will be run on all CIFs submitted for publication in IUCr journals (*Acta Crystallographica*, *Journal of Applied Crystallography*, *Journal of Synchrotron Radiation*); however, if you intend to submit to *Acta Crystallographica Section C* or *E* or *IUCrData*, you should make sure that full publication checks are run on the final version of your CIF prior to submission.

Publication of your CIF in other journals

Please refer to the *Notes for Authors* of the relevant journal for any special instructions relating to CIF submission.

PLATON version of 06/01/2024; check.def file version of 05/01/2024

Datablock 230429d - ellipsoid plot

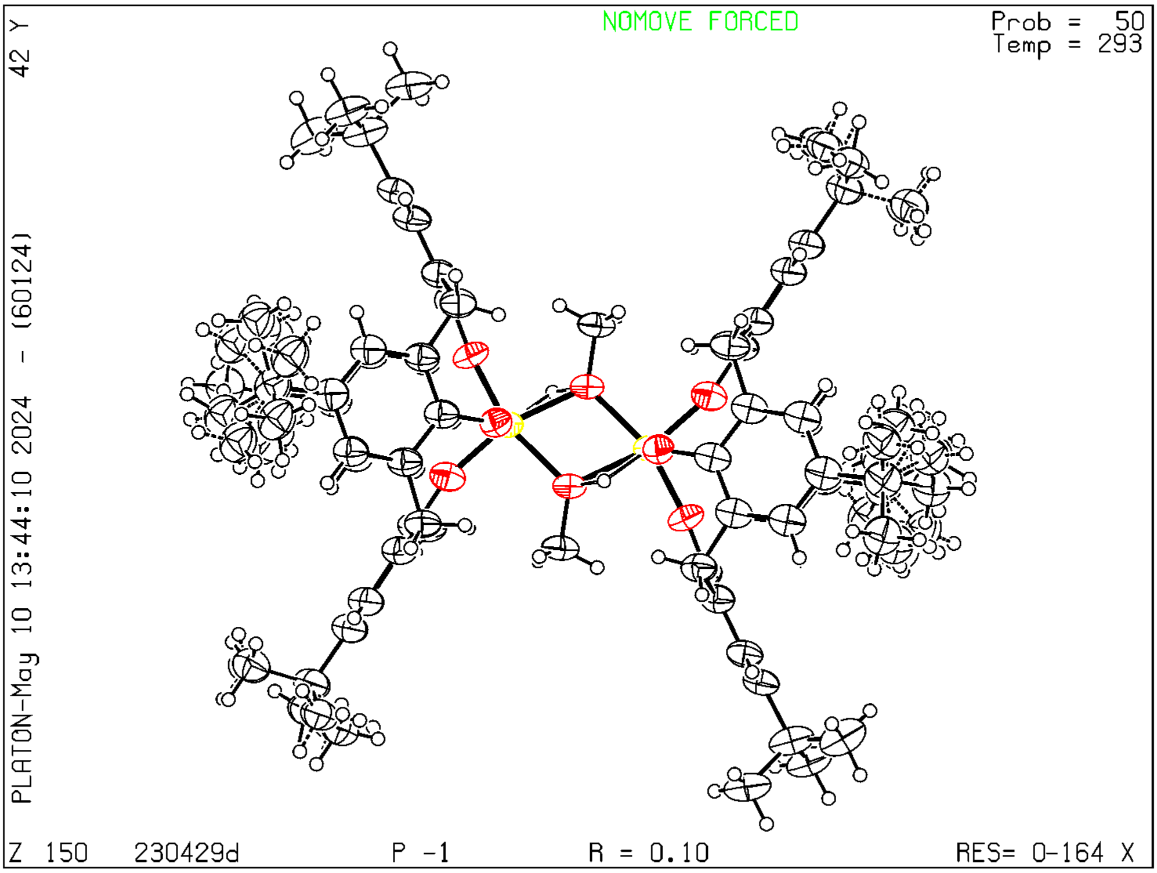

Supplement: Supplementary file 1 [file molecules-29-02566-s001.zip › molecules-3002188-supplementary/Supplementary Files-molecules-3002188-R2/Ti2-revised_cifreport.pdf]
